# miR-17-5p and miR-20a-5p suppress postoperative metastasis of hepatocellular carcinoma via blocking HGF/ERBB3-NF-κB positive feedback loop

**DOI:** 10.7150/thno.41365

**Published:** 2020-02-19

**Authors:** Dong-Li Liu, Li-Li Lu, Li-Li Dong, Yang Liu, Xin-Yu Bian, Bao-Feng Lian, Lu Xie, Duo Wen, Dong-Mei Gao, Ai-Wu Ke, Jia Fan, Wei-Zhong Wu

**Affiliations:** 1 Liver Cancer Institute, Zhongshan Hospital, Fudan University, Key Laboratory of Carcinogenesis and Cancer Invasion, Ministry of Education, Shanghai 200032, China; 2 Shanghai Center for Bioinformation Technology, Shanghai Academy of Science and Technology, Shanghai 201203, China; 3 Institute of Biomedical Sciences, Fudan University, Shanghai 200032, China

**Keywords:** hepatocellular carcinoma (HCC), miRNA-17-92 cluster, postoperative metastasis, HGF/ERBB3- NF-κB feedback loop, microenvironment remodeling

## Abstract

Dysregulation of microRNA (miRNA) is a frequent event in hepatocellular carcinoma (HCC), but little is known whether it is a bystander or an actual player on residual HCC metastasis during liver microenvironment remodeling initiated by hepatectomy.

**Methods:** The differently expressed miRNAs and mRNAs were identified from RNA-seq data. Western blot, qRT-PCR, fluorescence *in situ* hybridization, immunofluorescence and immunohistochemical were used to detect the expression of miRNA and mRNA in cell lines and patient tissues. The biological functions were investigated *in vitro* and *in vivo*. Chromatin immunoprecipitation, proximity ligation and luciferase reporter assay were used to explore the specific binding of target genes. The expression of HGF/ERBB3 signaling was detected by Western blot.

**Results:** In this study, HGF induced by hepatectomy was shown to promote metastasis of residual HCC cells. miR-17-5p and miR-20a-5p were confirmed to play inhibitory roles on HCC metastasis. And ERBB3 was found to be the common target of miR-17-5p and miR-20a-5p. HCC cells with lower levels of miR-17-5p and miR-20a-5p or higher level of ERBB3 were often more sensitive to response HGF stimuli and to facilitate metastatic colonization both *in vitro* and *in vivo* experimental systems. Furthermore, HGF reinforced ERBB3 expression by NF-κB transcriptional activity in a positive feedback loop. Of particular importance, HCC patients with lower levels of miR-17-5p and miR-20a-5p or higher level of ERBB3 had significantly shorter OS and PFS survivals after surgical resection.

**Conclusion:** miR-17-5p and miR-20a-5p could suppress postoperative metastasis of hepatocellular carcinoma via blocking HGF/ERBB3-NF-κB positive feedback loop and offer a new probable strategy for metastasis prevention after HCC resection.

## Introduction

Hepatocellular carcinoma (HCC) is one of the most lethal cancers in the world 1-3. Radical resection is currently used as a curative treatment for HCC patients 4. However, most patients already have intrahepatic, circulating system or distant micrometastasis at the time of surgery. Even if the gross tumor is completely removed, there may still be microscopic cancer lesions in the remaining liver that are difficult to be detected by naked eye and conventional imaging examination. So clinical resection cannot guarantee the complete eradication of tumors, and residual tumors were still confirmed in some cases after surgery 5. Previous studies show that about 60%-70% of surgical recipients will suffer from tumor recurrence and metastasis within 5 years 1,3. In our hospital, even those subclinical HCC patients with a single tumor less than 3 centimeters in diameter, their 5-year recurrence rate was up to 43.5% after hepatectomy 6,7. Unfortunately, traditional postoperative chemotherapy has low therapeutic efficacy and whether or not it actually works is still up for debate^8^. Therefore, unveiling the underlying mechanisms of postoperative relapse and intervention the metastasis at the molecular level become imperative in clinic.

Tumor metastasis is a complex process, which involved in both tumor cells and tumor microenvironment 9, as described by Stephen Paget in his “seed-and-soil” hypothesis 10. Most previous studies mainly focused on static tumor microenvironment remodeling with little attention to dynamic microenvironment changes after hepatectomy 11-13. Liver regeneration will be initiated immediately after hepatectomy, accompanied with a huge amount production of growth factors and cytokines known as 'cytokine storm' 14,15. Inevitably, the enormous changes of liver microenvironment are introduced to those residual HCC cells. However, the effects of this dynamic microenvironment remodeling postoperation on residual HCC cell colonization and metastasis are still unknown.

microRNAs (miRNAs), a class of endogenous small noncoding RNAs, can regulate comprehensive biological processes by changing the expression and translation of their target messenger RNA (mRNA) at post-transcriptional level 16,17. Several miRNAs have been validated to play vital roles for tumor progression by modulating epithelial-mesenchymal transition (EMT), reprograming energetic metabolism, promoting self-renewal and multi-lineage evolution, initiating tumor angiogenesis and remodeling tumor microenvironment 18-22.Up to now, it is unclear whether miRNA could regulate residual HCC cells metastasis induced by liver microenvironment dramatic changes after hepatectomy.

Here, we reported a novel function of miR-17-92 clusters, especially miR-17-5p and miR-20a-5p, in postoperative HCC metastasis. When overexpressed with ERBB3, HCC cells were more sensitive to ascending HGF stimuli and more apt to facilitate a metastatic clonal growth. By blocking HGF/ERBB3-NF-κB positive feedback loop, miR-17-5p and miR-20a-5p could suppress HCC metastasis significantly. These findings provide new insights of miR-17-92 clusters as tumor suppressors and offer a probable strategy for metastasis prevention after HCC resection.

## Materials and Methods

### Cell lines and animals

HCC cell lines, MHCC97L, MHCC97H, HCCLM3, HCCLM3-RFP (HCCLM3R), HCCLM3R-LM1-S4 and HCCLM3R-LnM1-S11, with step-wise lung and/or lymph node metastatic potentials in xenograft models were successfully established from one HCC patient in our institute. Low metastatic human HCC cell lines, HepG2 and Huh7, were purchased from American Type Culture Collection (Manassas, VA). These cell lines were cultured in DMEM medium, 10% fetal bovine serum (Hyclone, USA) and 1% penicillin-streptomycin (Invitrogen, USA). Male BALB/c nu/nu mice (4-6 weeks old; Shanghai Institute of Material Medicine, Chinese Academy of Science) were housed in specific pathogen free conditions. All animals received humane care according to the criteria outlined in the Guide for the Care and Use of Laboratory Animals prepared by the National Academy of Sciences and published by the National Institutes of Health (NIH publication 86-23, revised, 1985). The procedures for care and use of animals were approved by the Ethics Committee of Zhongshan Hospital of Fudan University (Shanghai, China). Vectors used in this study, cell transfection procedures, migration and invasion assays, luciferase reporter assays and* in vivo* assays for tumor metastasis are described in the Supplementary Materials and Methods.

### RNA and protein detection

RNA isolation, real-time PCR, Western blot, enzyme-linked immunosorbent assays (ELISA), proximity ligation assay (PLA), chromatin immunoprecipitation (ChIP), co-immunoprecipitation (CoIP), immunofluorescence analysis, immunohistochemistry procedures and analysis, as well as *in situ* hybridization, are described in the Supplementary Materials and Methods.

### *In vivo* assays for tumor metastasis

HepG2-miR-17-5p*^KD^*, HepG2-miR-20a-5p*^KD^* and HepG2-miR*^NC^* as well as HCCLM3-miR-17-5p*^OE^*, HCCLM3-miR-20a-5p*^OE^* and HCCLM3-miR*^NC^* orthotopic xenograft models were established in male athymic BALB/c nude mice (4-6 weeks old) for *in vivo* tumor invasion and metastasis analysis 23. Meanwhile, 1×10^5^ viable HCCLM3-ERBB3*^OE^*, HCCLM3-ERBB3*^NC^*, HCCLM3-HGF*^OE^*, HCCLM3-HGF*^NC^* cells were injected into mice via a lateral tail vein respectively. In partial hepatectomy model, mice were subjected to left lateral lobe resection 2 days before tail vein injections of HCCLM3-miR-17-5p*^OE^* or HCCLM3-miR-20a-5p*^OE^* cells. All mice were monitored once every 3 days and killed 6 weeks later. Living lung metastasis foci were evaluated by Quantum GX MicroCT Imaging System (PerkinElmer, Boston, MA). Bioluminescence imaging was performed using an IVIS Lumina K Series III, and image radiance values were normalized using Living Image (PerkinElmer, Boston, MA). Then, total metastatic foci were counted in paraffin embedded lungs under a microscope, as described previously 24. Tumor volume was calculated by Quantum GX MicroCT Imaging System (PerkinElmer, Boston, MA) or calculated as follows: V=ab^2^/2 (a and b representing the largest and smallest tumor diameters measured at necropsy 25. The metastases were classified into four grades on the basis of tumor cells present at the maximal section for each metastatic lesion: grade I, ≤20 tumor cells; grade II, 20-50 tumor cells; grade III, 50-100 tumor cells; and grade IV, >100 tumor cells 20.

### Patients and follow-up

One independent cohort including 104 paraffin-embedded HCC tissues was constructed from HCC patients undergoing curative resection in 2006. These patients were postsurgical follow-up until December 15, 2012. Histopathological diagnosis was based on World Health Organization criteria. Tumor grade was determined in accord with the classification proposed by Edmondson and Steiner. The Child-Pugh scoring system was used to assess liver function. Tumor stage was determined according to the tumor node metastasis classification system established by the 2010 International Union Against Cancer. A permitted use of human tissues in this study was approved by the research ethics committee of Zhongshan Hospital (Shanghai, China), and informed consent was obtained from each patient. Postsurgical patient surveillance was performed as previously described 26. Overall survival (OS) was defined as the interval between surgery and death or between surgery and the last observation point. For surviving patients, the data were censored at the last follow-up. Progression free survival (PFS) was defined as the interval between the surgery date and the date of any diagnosed relapse (intrahepatic recurrence and extrahepatic metastasis).

### Statistical analysis

Data were analyzed using GraphPad Prism 5 software. All data were expressed as mean ± standard deviation. Two-sided independent Student's t-test without equal variance assumption or the Wilcoxon signed-rank test was performed to analyze the differences in gene and miRNA expressed levels, tumor colonies and nodules, and *in vitro* luciferase assays. Spearman rank correlation coefficients were used for clinical associations study. The log-rank test was used to determine the statistical significance of the differences between progression-free survival curves and overall survival curves. The miRNA-target interactions were predicted by miRDB (http://www.mirdb.org/miRDB/. The pathway information was extracted from KEGG database (http://www.genome.jp/kegg/). R/Bioconductor software was used for all bioinformatics analysis. Results were considered statistically significant at p<0.05.

## Results

### HGF induced by hepatectomy promotes HCC metastasis

**Figure 1:**
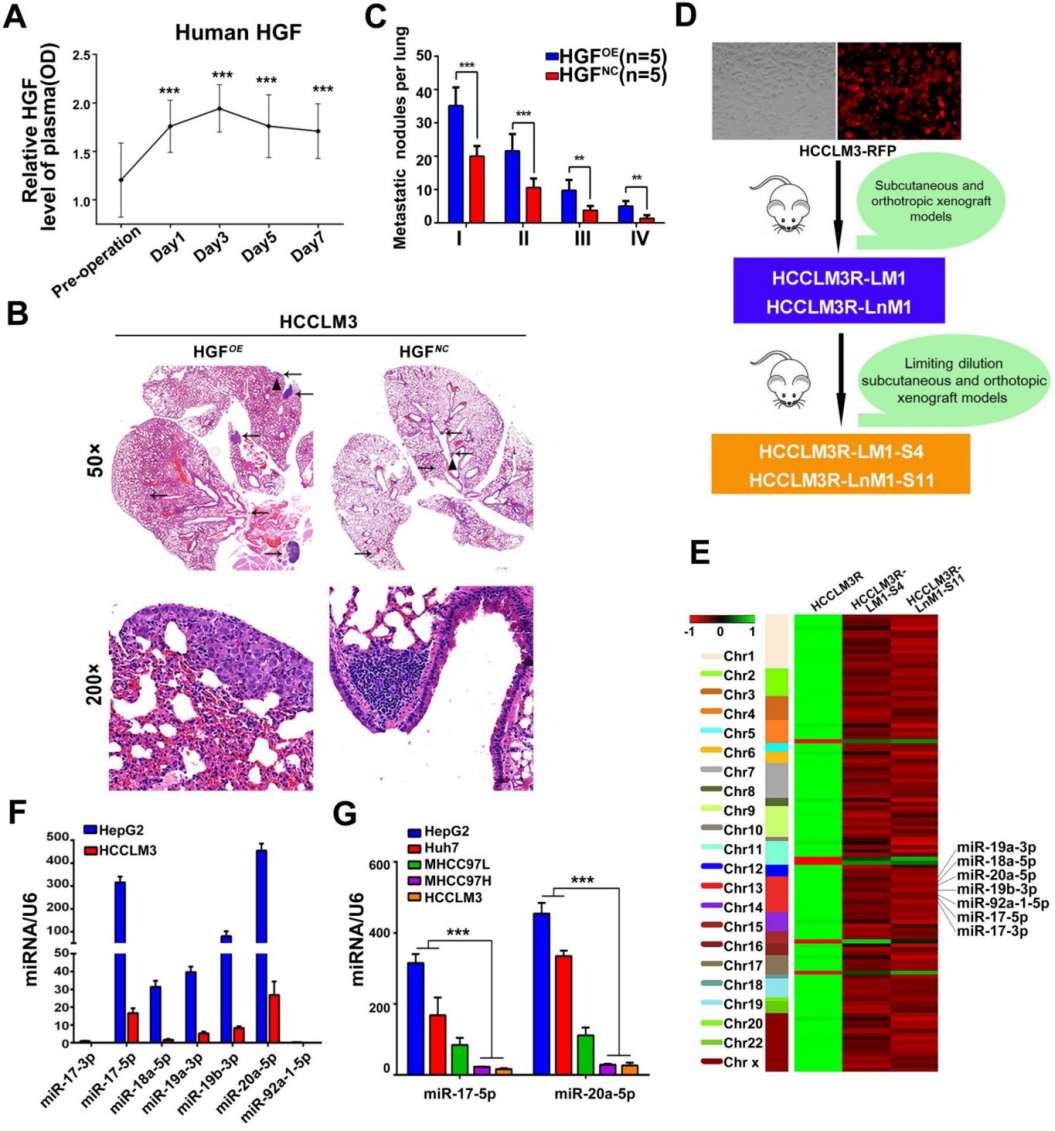
** HGF induced by hepatectomy promotes HCC metastasis and miR-17-92 cluster selected as metastatic candidates of HCC. (A)** Serum human HGF expression level in HCC patients pre- and post-operation are indicated by ELISA. Student's t-test. ***p<0.001. **(B)** H&E staining of metastatic nodules in lung in HGF overexpressed xenografts. The arrows and triangles all represented the lung metastatic foci with the HE staining, and of which, the triangle part was enlarged in its below figure. (top) magnification 50×, (bottom) magnification 200×. **(C)** The numbers of lung metastatic foci in HGF overexpressed xenografts. n=5. Student's t-test. **p<0.01; ***p<0.001. **(D)** The established roadmap of 2 metastatic cell lines. HCCLM3R-LM1 and HCCLM3R-LnM1 cell lines were established from lung/lymph node metastatic tissues of HCCLM3-RFP xenografts. HCCLM3R-LM1-S4 and HCCLM3R-LnM1-S11, 2 monoclonal cell lines, were derived from HCCLM3R-LM1 and HCCLM3R-LnM1 respectively, and exhibited much higher metastatic potentials than HCCLM3-RFP cells. **(E)** Differentially expressed miRNAs related to HCC metastasis screened by miRNA-seq, aligned according to their chromosome locations. The top color bar indicates expression levels, red showing down regulation in metastatic cells. **(F)** The endogenous levels of miR-17-92 cluster in HCCLM3 and HepG2 cells quantified by Real-time PCR analyses. U6 was included as a control. n = 3. **(G)** The endogenous levels of miR-17-5p and miR-20a-5p in HCCLM3, MHCC97H, MHCC97L, Huh7 and HepG2 cell lines quantified by Real-time PCR analyses. U6 was included as a control. n=3.Student's t-test. ***p<0.001. Data are mean ± SD and are representative of three independent experiments.

Numerous cytokines indispensable for hepatic growth and liver regeneration are immediately produced after hepatectomy. As a result, liver microenvironment to the residual HCC cells is astonishingly turned over. To definite the main cytokines responsible for liver regeneration, ELISA kits were used for a preliminary screening. Hepatocyte growth factor (HGF) rather than other growth factors, like epidermal growth factor (EGF), was observed an immediate outburst in the sera of forty HCC patients after tumor resection. The dynamic HGF levels increased immediately, reached the peak at the third day, then gradually decreased and maintained at relatively higher levels than preoperative levels at the 7^th^ day (Figure 1A, Figure S1A). These data suggest that HGF plays a pivotal physiological function in liver regeneration and is the important factor for liver microenvironment changes after surgery.

For another, HGF is confirmed as a major pathological player in tumor invasion and metastasis 27-29. We therefore investigated whether HGF promotes HCC invasive potentials. HCCLM3 cells treated with HGF did exhibit an increased migration compared with untreated cells (p<0.01) (Figure S1B-C). Similar results were observed in HepG2 cells (Figure S1D-E). Furthermore, lung metastatic foci of human HGF overexpressed HCCLM3 cells (HGF*^OE^*)were much more than these of negative control vector expressed cells (HGF*^NC^*)when injected via tail vein of nude mice (Figure 1B-C). In aggregate, these data indicate that HGF did promote HCC metastasis both *in vitro* and *in vivo*.

### Down-regulation of miR-17-5p and miR-20a-5p correlated with HCC metastasis and prognosis

Given that lung and lymph node are the very common metastatic sites for residual HCC cells, we previously established two HCC monoclonal cell lines with different organ-specific metastatic capabilities, HCCLM3R-LM1-S4 and HCCLM3R-LnM1-S11^23^. These two cells derived from HCCLM3R cells (HCCLM3-RFP) preferentially metastasize to lungs and regional lymph nodes, respectively (Figure 1D). All three of these cell lines have the same genetic background, but HCCLM3R-LM1-S4 and HCCLM3R-LnM1-S11 have higher metastatic potentials than HCCLM3R when gauged in xenograft models 23. To identify HCC metastasis-driving miRNAs and mRNAs, RNA sequencing (RNA-seq) were performed on the above three HCC cells. The differential expressed mRNAs and miRNAs can be achieved in https://www.ncbi.nlm.nih.gov/geo/query/acc.cgi?acc=GSE38945 (GEO: GSE38945). To narrow our candidates, a three-steps screening was designed. Firstly, all differentially expressed miRNAs and mRNAs were identified with log2 fold change absolute value ≥ 1 and FDR (false discovery rate) ≤ 0.05. Secondly, these miRNAs and mRNAs with the same expressed trends in HCCLM3R-LM1-S4 and HCCLM3R-LnM1-S11 cells compared to HCCLM3R cells were retained. By this step, 107 differentially expressed miRNAs and 296 mRNAs were obtained, representing the metastasis-related miRNAs and mRNAs in HCC (Table S1 and Table S2). Lastly, miR-17-92 cluster, mapped at the proximal loci on chromosome 13, were picked out for their expression in cluster and their negative association with the metastatic potential (Figure 1E). When assayed by RT-PCR, seven members of miR-17-92 cluster were remarkably down-regulated in high metastatic HCCLM3 cell compared to low metastatic HepG2 cells (Figure 1F). Therefore, we focused our efforts on exploration of this miRNA cluster.

**Figure 2:**
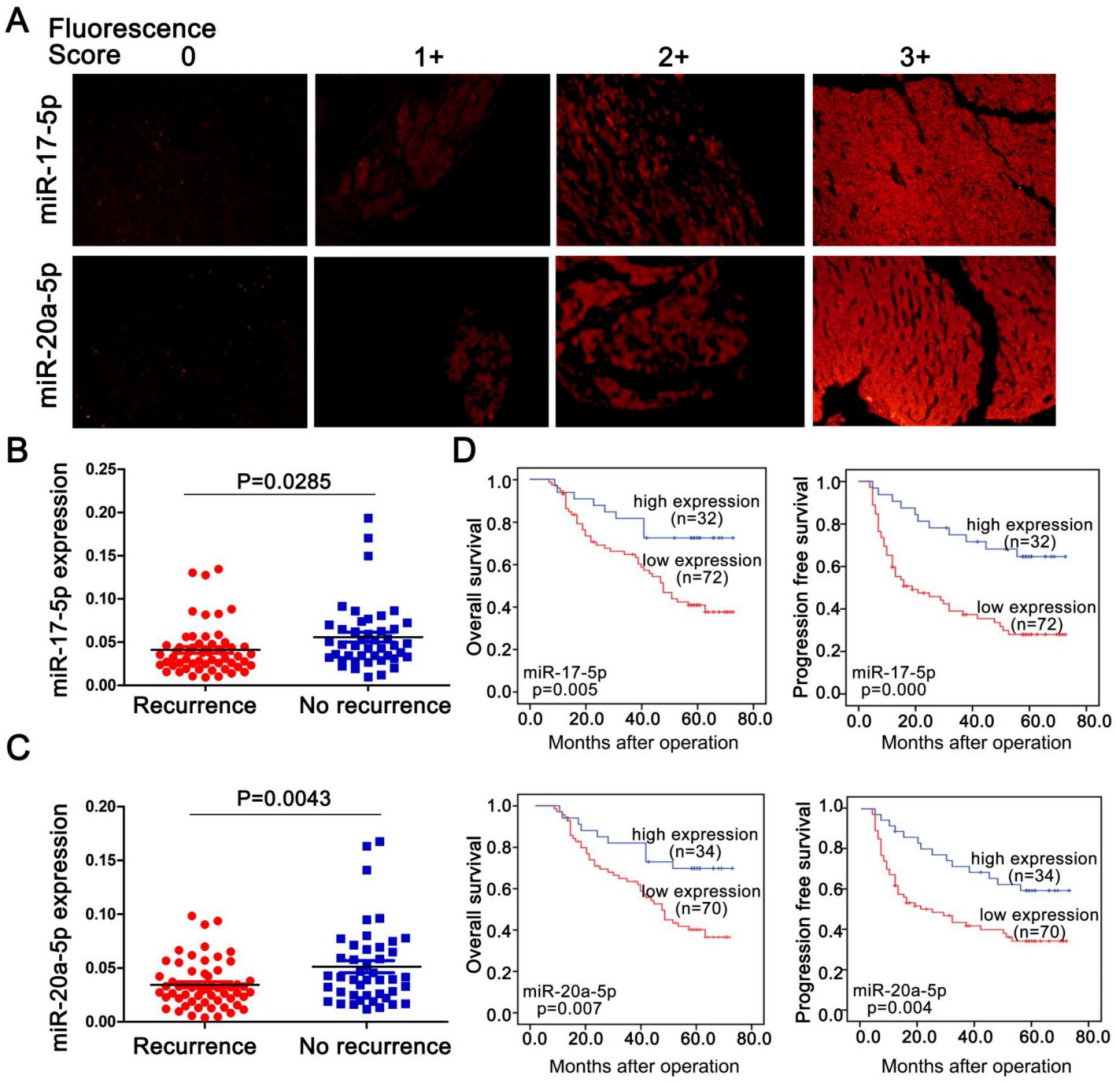
** Endogenous levels of miR-17-5p and miR-20a-5p inversely correlated with metastatic survivals in postoperative HCC patients. (A)** Representative images of miR-17-5p (top) and miR-20a-5p (bottom) by *in situ* hybridization on HCC tissue specimens. miR-17-5p and levels were scored as 0,1+,2+,3+ based on fluorescence intensity. Bars, 50μm. **(B)** Expression of miR-17-5p and **(C)** miR-20a-5p in HCC tissues with or without recurrence after surgical resection. **(D)** Kaplan-Meier's curves after stratified with miR-17-5p and miR-20a-5p levels. miRNA high expression (scores 2-3), miRNA low expression (scores 0-1). Data are mean ± SD and are representative of three independent experiments.

To explore a general significance of the cluster, miR-17-5p and miR-20a-5p, the two highest members were selected to further confirm in more HCC cell lines with different metastatic potentials. The levels of miR-17-5p and miR-20a-5p in three high metastatic HCC cell lines (MHCC97L, MHCC97H and HCCLM3) were significantly decreased compared to the low metastatic cell lines, HepG2 and Huh7 (Figure 1G). Next, the expression of these two miRNAs was measured in 104 postoperative HCC samples by miRNA *in situ* hybridization (Figure 2A). miR-17-5p and miR-20a-5p levels in HCC patients with recurrence were lower than those without recurrence (Figure 2B-C). The outcomes of miR-17-5p*^high^* patients were significantly better than those of miR-17-5p*^low^* group (Figure 2D). Similarly, both overall survival (OS) and progression free survival (PFS) were significantly prolonged in miR-20a-5p*^high^* patients compared to patients with miR-20a-5p*^low^* expressed (Figure 2D). The analysis of TCGA database also reviewed that those miR-17-5p*^ high^* or miR-20a-5p*^high^* patients achieved a better OS (p=0.023 and p=0.027, respectively; Figure S4G-H). Thus, miR-17-5p and miR-20a-5p levels were down-regulated in HCCs, particularly in patients with postoperative metastasis, and these findings indicate a potential role for miR-17-5p and miR-20a-5p in HCC progression.

### miR-17-5p and miR-20a-5p deficiency promote HCC metastasis *in vitro* and in nude mice models

**Figure 3:**
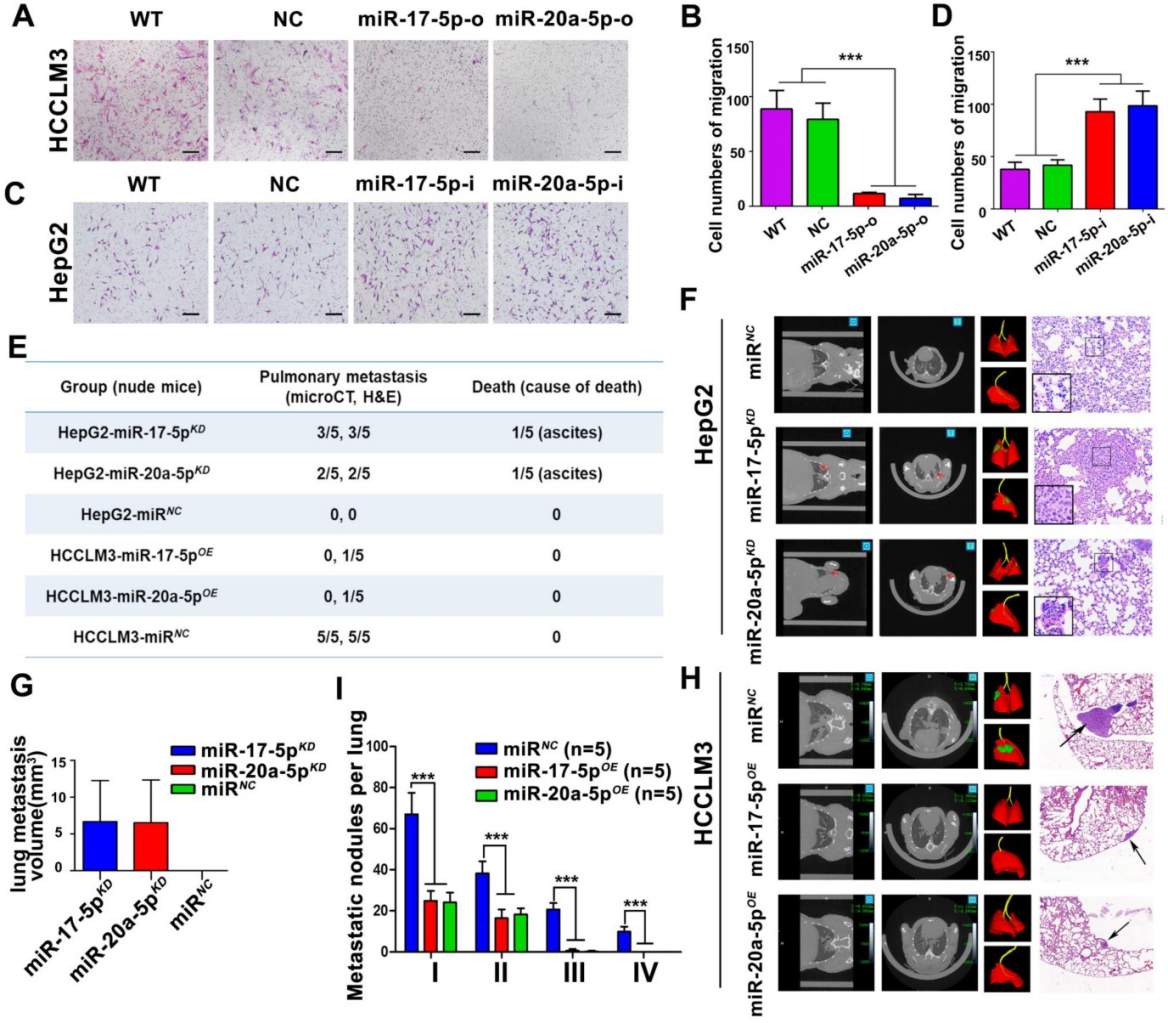
** Suppressive effects of miR-17-5p and miR-20a-5p on HCC metastasis *in vitro* and xenograft models. (A&C)** Representative motility images. Bars, 200 μm. **(B&D)** Motility of HCCLM3 and HepG2 cells with indicated treatment. n=3. WT, NC, miR-17-5p-o, miR-20a-5p-o, or miR-17-5p-i, miR-20a-5p-i were defined as none, irrelevant oligonucleotides, miR-17-5p mimics, miR-20a-5p mimics, miR-17-5p inhibitors and miR-20a-5p inhibitors transfected cells, respectively. Student's t-test. ***p<0.001. **(E)** miR-17-5p/miR-20a-5p overexpressed HCCLM3 cells and miR-17-5p/miR-20a-5p knockdown HepG2 cells was *in situ* injected in Balb/c nude mice. The table shows the pulmonary metastasis and survival rate among different xenograft mice group by micro-spiral CT scan after 6 weeks. **(F&H)** Representative CT scanning images of metastatic nodules in lungs in indicated xenograft mice. The first panel: Coronal scanning images; The second panel: Cross-sectional scanning images; The third panel: Three-dimensional images. Red, normal lung tissue; Green, metastatic foci. The forth panel: Representative images of hematoxylin eosin (H&E) staining of metastatic nodules in lungs from indicated xenograft mice. **(G)** Tumor volumes of lung metastatic foci in indicated HepG2 xenografts. **(I)** The numbers of lung Metastatic foci in indicated HCCLM3 xenografts. N=5. Student's t-test. ***p<0.001. Data depicted as the mean ± SD from three independent experiments.

In order to explore the biological significances of miR-17-5p and miR-20a-5p, loss- and gain-of-function studies were investigated using a transient transfection strategy with miR-17-5p and miR-20a-5p mimics or inhibitors. In motility assays, HCCLM3 cells were reduced more than 6.1 and 5.1 folds, respectively, by miR-17-5p-o and miR-20a-5p-o compared with the capabilities of control cells (p<0.001; p<0.001; Figure 3A-B). In invasion assays, HCCLM3 cells were reduced more than 4 and 3.9 folds after miR-17-5p-o and miR-20a-5p-o treatments, respectively (p<0.001; p<0.001; Figure S2B-D). In contrast, the numbers of migratory HepG2 cells increased more than 2.2 and 2.4 folds after miR-17-5p-i and miR-20a-5p-i treatments compared with non-transfected counterparts, respectively (p<0.001, p<0.001; Figure 3C-D). And the numbers of invasive HepG2 cells increased more than 2.6 and 2.5 folds with miR-17-5p-i and miR-20a-5p-i treatments compared with control cells (p<0.001, p<0.001; Figure S2C-E). These results suggest that miR-17-5p and miR-20a-5p negatively regulate tumor migration and invasion of HCC.

After transplanted with stably miR-17-5p and miR-20a-5p overexpressed (OE) or knockdown (KD) human HCC cells (Figure S2A), a series of orthotopic xenograft models were successfully established in Balb/c nude mice. After 6 weeks, 3 of 5 mice derived from HepG2-miR-17-5p*^KD^* xenografts, 2 of 5 mice derived from HepG2-miR-20a-5p*^KD^* xenografts developed obvious pulmonary metastasis foci when detected by micro-spiral CT scan, while no pulmonary metastasis occurred in HepG2-miRNA-negative control cells (HepG2-miR*^NC^*) mice (Figure 3E-F). More important, one in HepG2-miR-17-5p*^KD^* mice and one in HepG2-miR-20a-5p*^KD^*mice died of ascites, respectively (Figure 3E). The mean lung metastasis sizes of HepG2-miR-17-5p*^KD^* and HepG2-miR-20a-5p*^KD^* xenografts were 6.65 mm^3^ and 6.52 mm^3^, respectively, which are significantly larger than HepG2-miR*^NC^* xenografts (Figure 3G). The pulmonary metastasis rate in HCCLM3-miR*^NC^* mice was 100% (5 of 5), while no metastasis was found in HCCLM3-miR-17-5p*^OE^* mice and HCCLM3-miR-20a-5p*^OE^* mice by micro-CT scanning (Figure 3H). One in HCCLM3-miR-17-5p*^OE^* mice and one in HCCLM3-miR-20a-5p*^OE^*were detected pulmonary micro-metastases using histological examinations (Figure 3E). The numbers of metastatic nodules in HCCLM3-miR*^NC^* mice were significantly greater than those in HCCLM3-miR-17-5p*^OE^* mice and HCCLM3-miR-20a-5p*^OE^* mice (Figure 3H-I). All these results suggest that stably forced expressions of miR-17-5p or miR-20a-5p conspicuously suppressed HCC lung metastasis, and deficiencies of these two miRNAs promote tumor metastasis *in vivo* system.

### ERBB3 is a common target of miR-17-5p and miR-20a-5p

**Figure 4:**
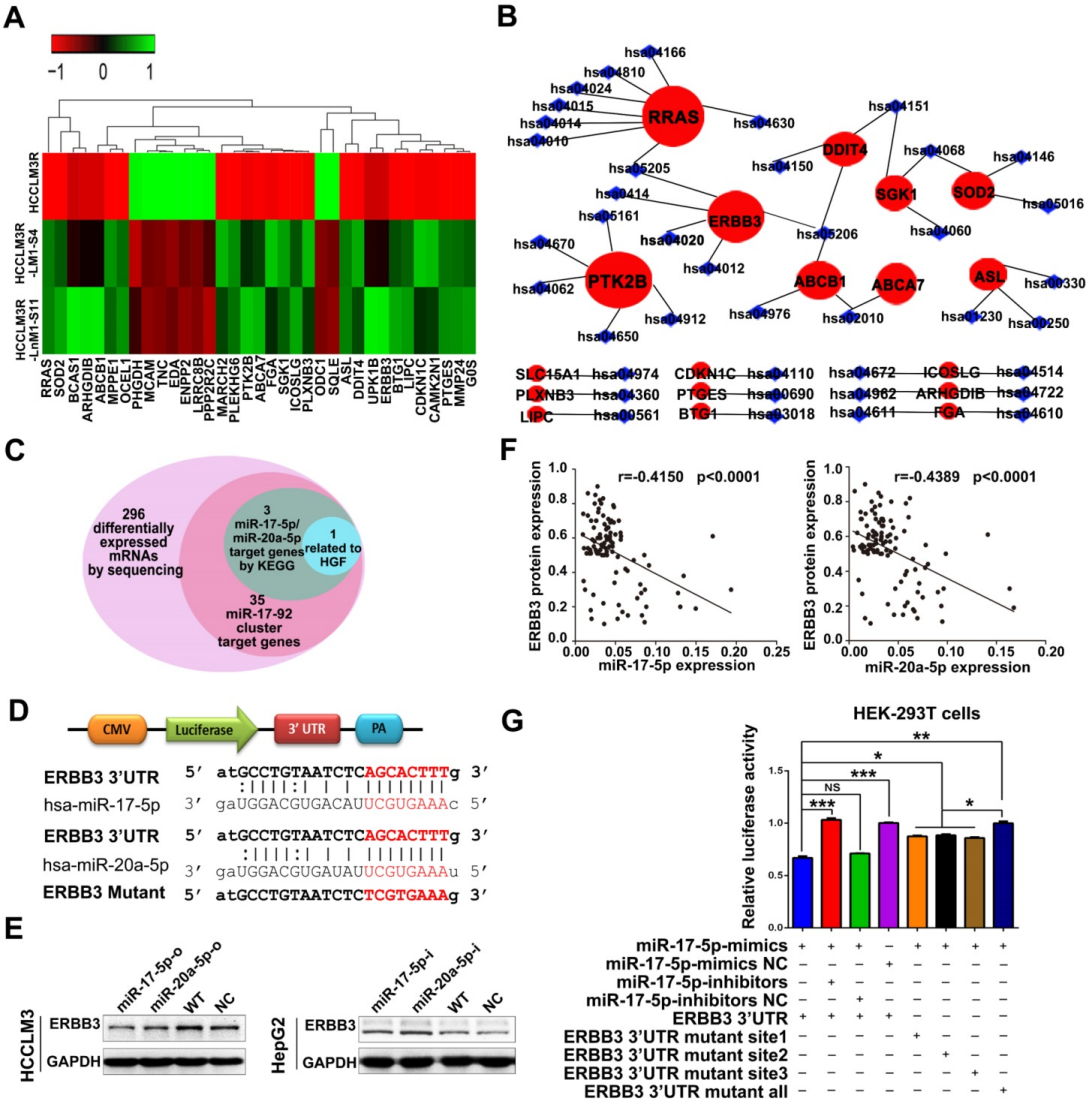
** ERBB3 identified as a direct downstream target of miR-17-5p and miR-20a-5p. (A)** Hierarchical clustering of differentially expressed mRNAs in HCCLM3R, HCCLM3R-LM1-S4 and HCCLM3R-LnM1-S11 cells. **(B)** Predicted target genes and their involved signaling pathways of miR-17-92 cluster, the hallmark target genes with the most pathways involved are shown in big red circles. **(C)** Venn diagrams of target genes of miR-17-5p and miR-20a-5p. **(D)** Schematic diagram of the dual luciferase miRNA target reporters. The wild-type seed sequence (red, upper two panels) and the mutant (red, lower panel) at 3'UTR of ERBB3 were cloned into pmirGLO dual luciferase reporter system. **(E)** Protein levels of ERBB3 in HCCLM3 and HepG2 cells after indicated treatments. **(F)** Scatter plots of ERBB3, miR-17-5p and miR-20-5p levels in tumor tissues of HCC patients. **(G)** Luciferase activities of ERBB3 reporter in miR-17-5p or miR-20a-5p treated HEK-293T cells. Student's t-test. *p<0.05; **p<0.01; ***p<0.001. Data are mean ± SD and are representative of three independent experiments.

As previously mentioned, miR-17-5p and miR-20a-5p were obtained from 107 differential expression miRNAs by our RNA-seq analysis. Theoretically, their corresponding target genes would be also differentially expressed. We thus inferred that their target genes could be in the 296 differentially expressed mRNAs from our previous RNA-seq and we used several bioinformatics methods to help identify the target genes using the following steps. (1) Thirty-five mRNAs are predicted target genes of seven members of miR-17-92 cluster by miRDB analysis (Figure 4A). (2) RRAS, PTK2B and ERBB3 were found as the three hallmarks in the whole predicted gene network of miR-17-5p and miR-20a-5p when analyzed by KEGG pathway analysis (Figure 4B). (3) miR-17-5p and miR-20a-5p have homologous sequences, suggesting they may target the same genes. (4) What is more, the target genes should be related to HGF or the downstream signaling pathways of HGF based on our previous study (Figure 4C). We were delighted to find that only ERBB3 meet the above criteria. In agreement with this notion, the complementary sequences of miR-17-5p and miR-20a-5p were identified in the 3'-UTR (untranslated region) of ERBB3 mRNA by TargetScan (Figure 4D). Western blot demonstrated that overexpression of miR-17-5p and miR-20a-5p significantly reduced ERBB3 levels in HCCLM3 cells (Figure 4E left). In contrast, miR-17-5p and miR-20a-5p knockdown dramatically enhanced expression of ERBB3 in HepG2 cells compared to control cells (Figure 4E right). In addition, a significant inverse correlation between miR-17-5p or miR-20a-5p and ERBB3 protein level was observed in HCC samples (p<0.0001, p<0.0001; Figure 4F). However, the mRNA level of ERBB3 was not affected by these two miRNAs (Figure S3A-B), indicating that miR-17-5p and miR-20a-5p suppressed ERBB3 expression at the post-transcriptional level. To test a direct role of miR-17-5p and miR-20a-5p on ERBB3 expression, 3 binding sites at conserved 3'UTR region of the gene were predicted and identified by luciferase reporter assays respectively (Table S3). The results showed that the luciferase activities were significant decreased in all three reporters with wild-type binding sequences, but not with mutants (Figure 4G, Figure S3C). Overall, ERBB3 was confirmed as a common direct target of miR-17-5p and miR-20a-5p.

### Elevated expression of ERBB3 enhances HCC metastasis and its direct association with clinicopathologic characteristics

**Figure 5:**
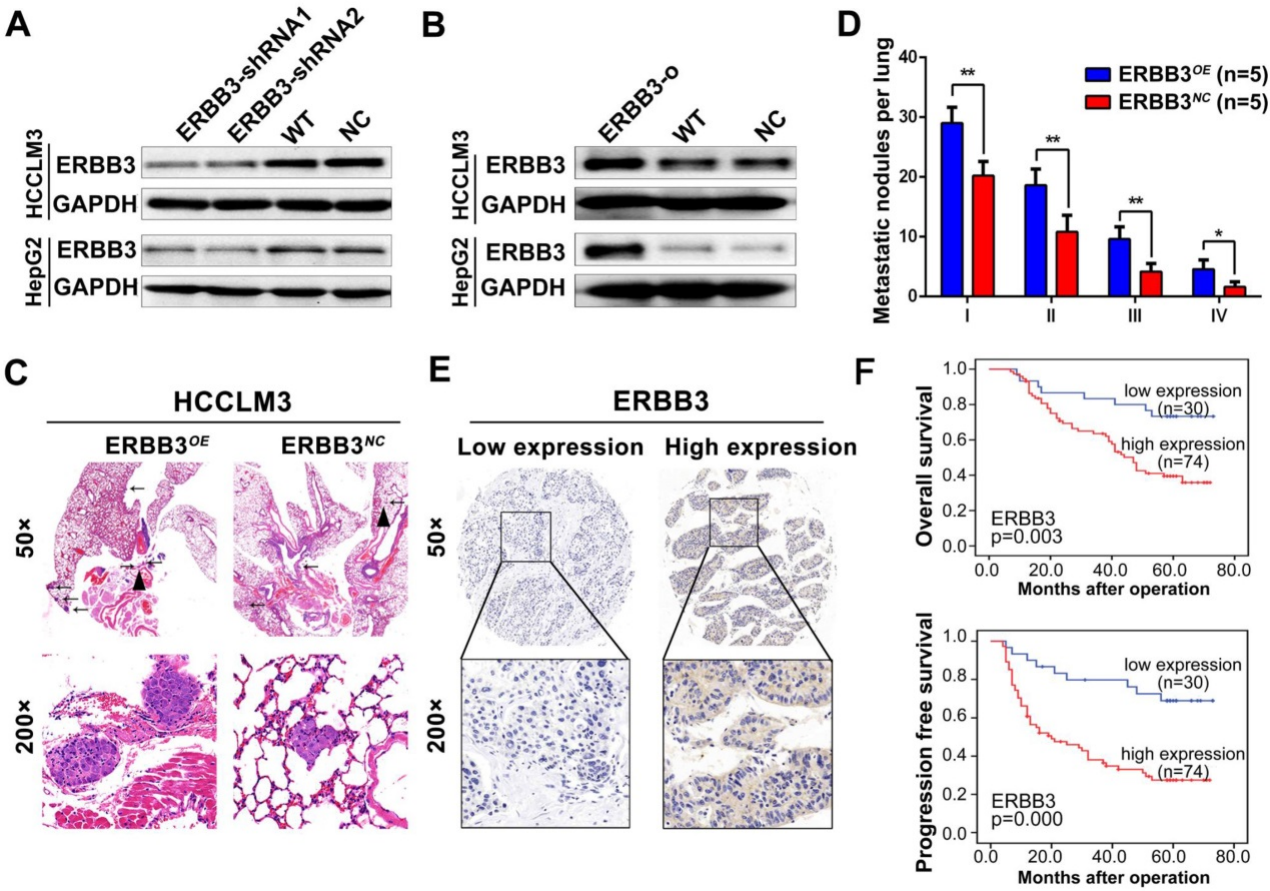
** Tumor metastasis enhanced by forced ectopic ERBB3 expression. (A&B)** ERBB3 protein levels in HCCLM3 and HepG2 cells transfected ERBB3 shRNA and overexpression vectors. **(C)** H&E staining of metastatic nodules in lung in ERBB3 overexpressed xenografts. The arrows and triangles all represented the metastatic nodules in lung with the HE staining, and of which, the triangle part was enlarged in its below figure. (top) magnification 50×, (bottom) magnification 200×. **(D)** The numbers of lung metastatic foci in ERBB3 overexpressed xenografts. N=5. Student's t-test. *p<0.05; **p<0.01. **(E)** Representative images of ERBB3 level in tumor tissues of HCC patients, (top) magnification 50×, (bottom) magnification 200×. **(F)** ERBB3 correlation to postoperative survivals of HCC patients: Kaplan-Meier's curves of 104 HCC patients after stratified by ERBB3 levels were used for depicting overall survival and progression free survival. Data depict the mean ± SD and are representative of three independent experiments.

As previously described, ERBB3 might serve as an oncogene in HCC progression 30,31. We tested the effect of ERBB3 on migratory and invasiveness of HCC cells. ERBB3-shRNA (ERBB3-shRNA1, ERBB3-shRNA2) or ERBB3-o (ERBB3-overexpression) plasmids were transfected into HCCLM3 and HepG2 cells. Expression of ERBB3 was confirmed by Western blot (Figure 5A-B). As expected, both migration and invasion capabilities were significantly inhibited in HCC cells, and remarkably promoted in ERBB3-o cells compared to control cells (Figure S3D-F). Similarly, the lung metastatic nodules in ERBB3*^OE^* xenografts were markedly increased compared with ERBB3*^NC^*counterparts after injecting HCC cells into nude mice (Figure 5C-D).

We examine the expression of ERBB3 using tissue microarray comprised of 104 HCC patients. The clinicopathologic characteristics of ERBB3 were described in Table S4. We found that ERBB3 existed in both cell membrane and nuclei, and its positive rate was 71.1% (74/104) in all HCC patients (Figure 5E). Moreover, we observed that stronger ERBB3 expression was correlated with AFP (p=0.023), GGT (p=0.012), tumor size (p=0.011), tumor thrombus (p=0.047), and TNM stage (p=0.007) (Table S4). Kaplan-Meier analysis showed that the outcomes of ERBB3*^high^* patients were significantly shorter than those of ERBB3*^low^* patients (log-rank, p<0.01; Figure 5F). Further analysis of TCGA database confirmed patients with high level of ERBB3 had worse OS (p=0.077) and RFS (p=0.011; Figure S4E-F). Taken together, all results raised from our *in vitro* system, xenograft models and clinical tissues imply that ERBB3 is an important metastatic factor during HCC progression.

### miR-17-5p and miR-20a-5p negatively regulate EMT via modulation of ERBB3 and their prognostic value for HCC patients

**Figure 6:**
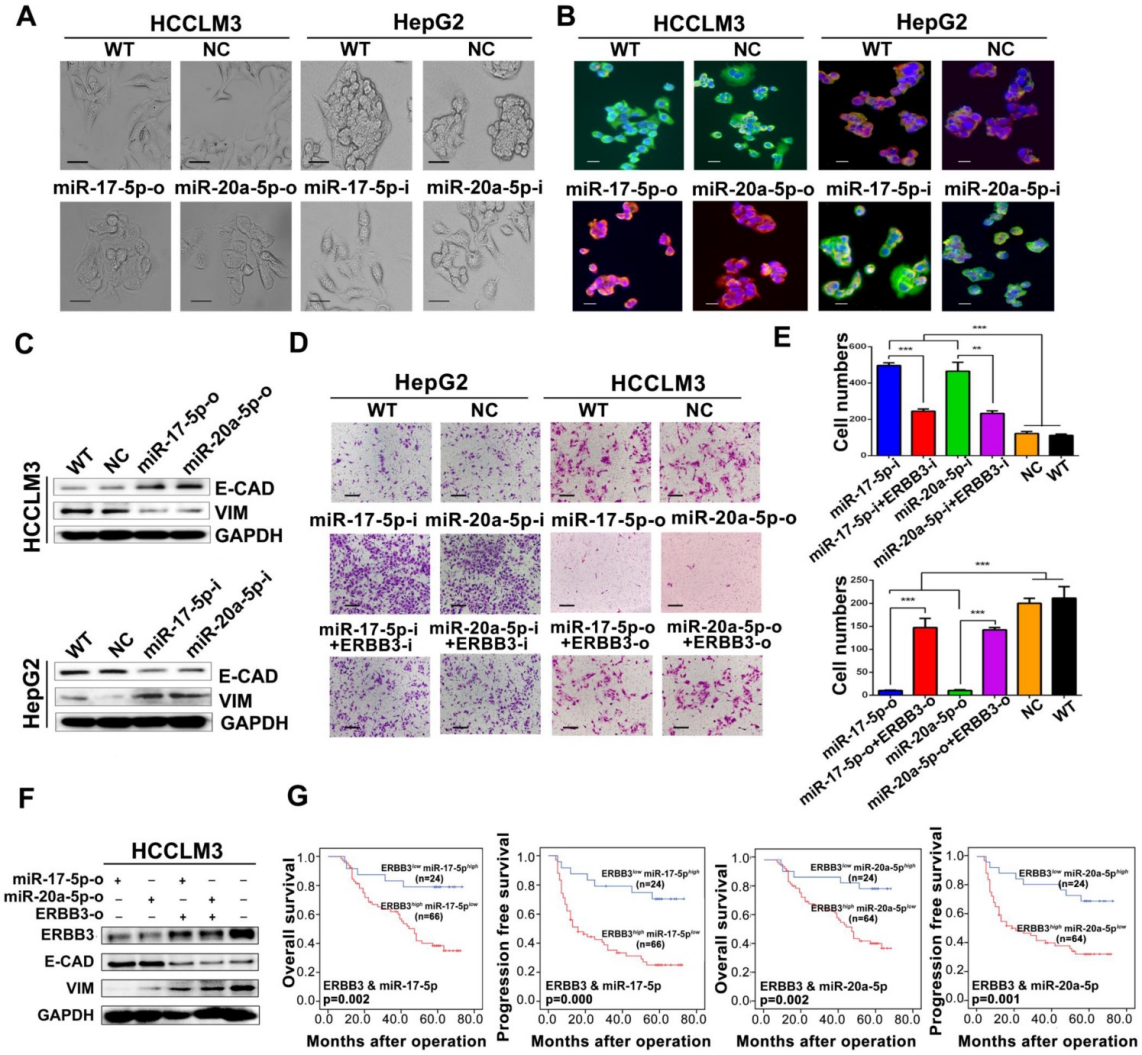
** EMT and patient survival negatively regulated by miR-17-5p and miR-20a-5p via ERBB3. (A)** Typical EMT morphogenesis in HCCLM3 and HepG2 cells after indicated treatments. Bars: HCCLM3,100 μm; HepG2 cell, 200 μm. **(B)** Expression of Vimentin(green) and E-cadherin(red) in HCCLM3 cells and HepG2 cells with indicated treatments by immunofluorescence. Bars, 200 μm. **(C)** E-cadherin (E-CAD) and Vimentin (VIM) levels of miR-17-5p and miR-20a-5p treated HCCLM3 and HepG2 cells. **(D)** Representative migration images of HepG2 and HCCLM3 cells after indicated treatments. Bars, 200 μm. **(E)** Statistical migration results of HepG2 and HCCLM3 cells with indicated treatment. n=3. Student's t-test. **p<0.01; ***p<0.001. **(F)** The protein levels of E-cadherin (E-CAD) and Vimentin (VIM) in HCCLM3 cells after indicated treatments. **(G)** The combined effects of ERBB3 and miR-17-5p/miR-20a-5p on postoperative survivals in HCC patients. Data depict the mean ± SD and are representative of three independent experiments.

During above studies, a typical morphological change of HCCLM3 and HepG2 cells was noted after treatment with miR-17-5p and miR-20a-5p mimics or inhibitors. Compared with control cells, HCCLM3 cells assumed a condensed and cobblestone-like morphology after treated with miR-17-5p-o or miR-20a-5p-o (Figure 6A left), whereas HepG2 cells exhibited a scattered, spindly or star-like morphology after treated with miR-17-5p-i or miR-20a-5p-i (Figure 6A right). These phenomena suggest that both miR-17-5p and miR-20a-5p were probably involved in the regulation of epithelial-mesenchymal transition (EMT) of HCC. Naturally, the expression of Vimentin and E-Cadherin, two vital proteins of EMT, were analyzed after miR-17-5p or miR-20a-5p manipulation by immunofluorescence. Vimentin (green staining) were expressed much sharper in miR-17-5p-i and miR-20a-5p-i treated HepG2 cells, while E-Cadherin (red staining) was at higher level in miR-17-5p-o or miR-20a-5p-o treated HCCLM3 cells (Figure 6B). Simultaneously, the western blot assay identified that the protein levels of E-cadherin and Vimentin were significantly increased and decreased, respectively, in miR-17-5p-o or miR-20a-5p-o treated HCCLM3 cells, and vice versa (Figure 6C). These observations indicate that miR-17-5p and miR-20a-5p can suppress the EMT of HCC.

A rescue assay was performed to investigate that ERBB3 is a critical mediator of miR-17-5p and miR-20a-5p in HCC EMT and metastasis. Notably, the promoting effects of miR-17-5p-i and miR-20a-5p-i on HCC migration were largely compromised in ERBB3-shRNA co-transfected HepG2 cells (Figure 6D left , Figure 6E upper), and greatly restored in ERBB3 rescued HCCLM3 cells when co-transfected with ERBB3 overexpression vector lacking of miR-17-5p and miR-20a-5p binding sites (Figure 6D right, Figure 6E lower). In addition, increased E-cadherin and decreased Vimentin levels in miR-17-5p-o or miR-20a-5p-o treated HCCLM3 cells were significantly reversed respectively when co-transfected with rescued ERBB3 overexpression vector (Figure 6F). In clinic, both OS and PFS were significantly decreased in ERBB3*^high^* miR-17-5p*^low^* patients than those of ERBB3*^low^* miR-17-5p*^high^* patients. A similar result was also found in ERBB3*^high^* miR-20a-5p*^low^* patients relative to ERBB3*^low^* miR-20a-5p*^high^* individuals (Figure 6G). These findings indicate that these two miRNAs negatively regulate the EMT of HCC via modulation of ERBB3 and the joint use of miRNAs and ERBB3 has prognostic value.

### HGF regulates ERBB3 expression, activates its downstream pathways, stimulates the transcriptional activity of NF-κB and enhances the heterodimer of ERBB3 and MET

**Figure 7:**
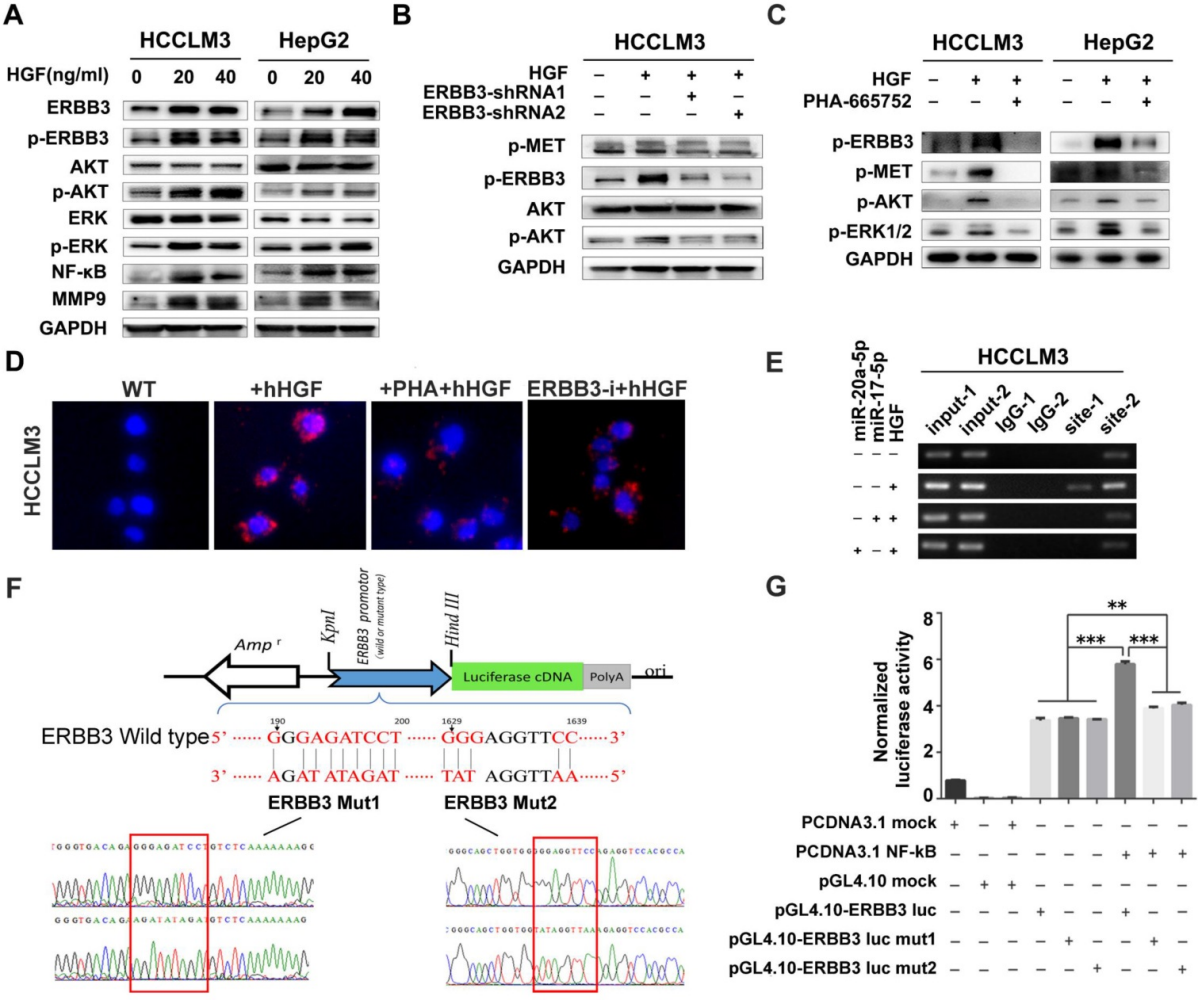
** ERBB3 levels directly induced by HGF. (A)** AKT and ERK signaling regulated by HGF in HCC cells. **(B)** AKT signaling regulated by HGF in ERBB3 knocked-down HCCLM3 cells. **(C)** Abolishment of PHA-665752 on HGF initiating AKT and ERK signaling. **(D)** Reversed effects of ERBB3 knocked-down or PHA-665752 treatment on ERBB3/Met interaction (red fluorescence, PLA) in HGF primed HCCLM3 cells. Bars, 50μm. **(E)** NF-κB binding on ERBB3 promotor regulated by HGF stimuli. **(F)** The schematic cartoons to show the dual luciferase ERBB3 reporters with wild-type and 2 mutant designs. **(G)** Luciferase activities regulated by NF-κB with wild-type or mutated ERBB promotor. Student's t-test. **p<0.01; ***p<0.001. Data are mean ± SD and are representative of three independent experiments.

A series of signaling pathways events including PI3K/AKT and MAPK signaling could be activated upon HGF stimuli. Therefore, we investigated the total and phosphorylated proteins of AKT (p-AKT) and ERK (p-ERK) by Western blot after HCCLM3 cells treated with 20 ng/ml or 40 ng/ml HGF for 0, 10, 20, 30 and 60 minutes, or with 10 ng/ml NRG-1 as a positive control. Both p-AKT and p-ERK but not total protein were significantly increased after a 30-minutes treatment (Figure S4A). Notably, the phosphorylated ERBB3 (p-ERBB3) were simultaneously up-regulated after 20 ng/ml or 40 ng/ml HGF treatments for 30 minutes and also total ERBB3 gradually increased starting from 30 minutes (Figure 7A left). Similar results were observed in HepG2 cells upon HGF stimuli (Figure 7A right). Moreover, NF-κB and its downstream target, MMP9, were also dramatically induced in both HGF treated HCCLM3 and HepG2 cells (Figure 7A). Next, we analyzed the effect of altered ERBB3 expression on HGF downstream signaling. We transfected HCCLM3 cells with two stable ERBB3-shRNAs. After treated with 20 ng/ml HGF for 30 minutes, the phosphorylated ERBB3 and AKT in both ERBB3-shRNAs transfected cells were significantly dampened (Figure 7B). These results suggest that HGF upregulates ERBB3 expression, activates its downstream signaling and promotes NF-κB transcriptional activity.

It is reported that ERBB3 could initiate downstream signaling when forming a heterodimer with other EGFR member 32,34. In our study, we found that phosphorylated proteins of ERBB3 and MET were simultaneously up-regulated in both HGF-treated HepG2 and HCCLM3 cells, but notably attenuated by MET inhibitor, PHA-665752 (Figure 7C), and ERBB3-shRNAs(Figure 7B).We next investigated the heterodimer formation of ERBB3 with MET upon HGF stimuli using a proximity ligation assay (PLA). The protein interacting signals (red spots) between ERBB3 and MET were significantly increased after treated with 20 ng/ml HGF, and thereafter largely blocked in either 10μM PHA-665752 treated or ectopic ERBB3-shRNAs expressed HCCLM3 cells (Figure 7D, Figure S4B). Consistent with the above finding, the protein interaction of MET with ERBB3 by CoIP were found in all indicated cells, especially in HGF-treated HCCLM3^WT^ cells (Figure S4C). These results demonstrate that HGF promotes ERBB3 interaction with MET and form ERBB3/MET heterodimer in HCC cells.

### ERBB3 is a direct transcriptional target of NF-κB in HCC cells

As both ERBB3 and NF-κB levels were significantly increased by exogenous stimuli of HGF, we investigated whether HGF enhanced ERBB3 mRNA transcription directly by NF-κB. Sequence analysis showed that two putative NF-κB binding sites (NF-κB-1 and NF-κB-2) existed in ERBB3 promoter regions. After chromatin immunoprecipitation (ChIP) by specific anti-NF-κB antibody, both NF-κB-1 and NF-κB-2 binding DNA sequences were successfully amplified by PCR primers listed in Table S5. Most importantly, a significant amplified band in NF-κB-2 but not in NF-κB-1 binding site was found in HGF priming cells compared to control cells (Figure 7E), suggesting that NF-κB-2 sequence might act as distal enhancer of ERBB3 expression upon HGF stimuli.

To test the transcriptional activities of NF-κB-1 and NF-κB-2 on ERBB3 expression, a serial vectors with wild-type or mutated binding sequence (Mut1 and Mut2) of ERBB3 were constructed (Figure 7F). Luciferase reporter assays revealed that NF-κB did promote ERBB3 transcriptional activities in both wild-type sequences, but not in their corresponding mutants (Figure 7G), which suggest that NF-κB is a direct upstream modulator on ERBB3 transcription.

### miR-17-5p and miR-20a-5p suppress HCC metastasis by blocking HGF/ERBB3-NF-κB positive feedback loop after hepatectomy

**Figure 8:**
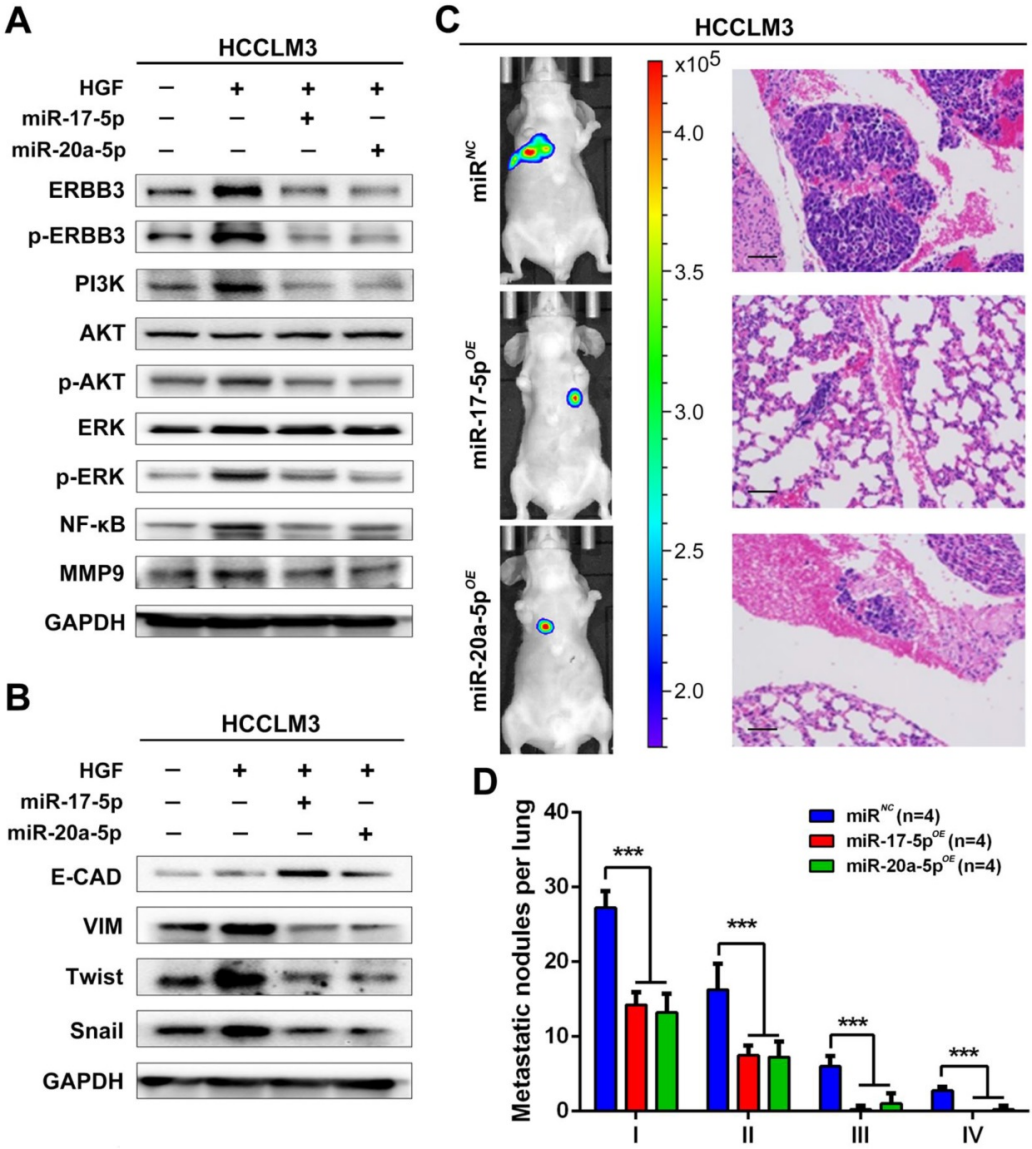
** miR-17-5p and miR-20a-5p suppress HCC metastasis by blocking HGF/ERBB3-NF-κB positive feedback loop after hepatectomy. (A)** AKT and ERK signaling regulated by HGF in miR-17-5p or miR-20a-5p mimic transfected cells. **(B)** EMT regulated by HGF in miR-17-5p or miR-20a-5p mimic transfected cells. **(C)** Bioluminescence images (left panel) and H&E staining (right panel) of lung metastatic nodules in miR-17-5p or miR-20a-5p overexpressed xenograft mice. Bars, 100μm. **(D)** The numbers of lung metastatic foci in partial liver resection xenograft mice. n=4. Student's t-test. ***p<0.001. **(E)** Hypothesis illustration of the study. Data are mean ± SD and are representative of three independent experiments.

**Scheme 1:**
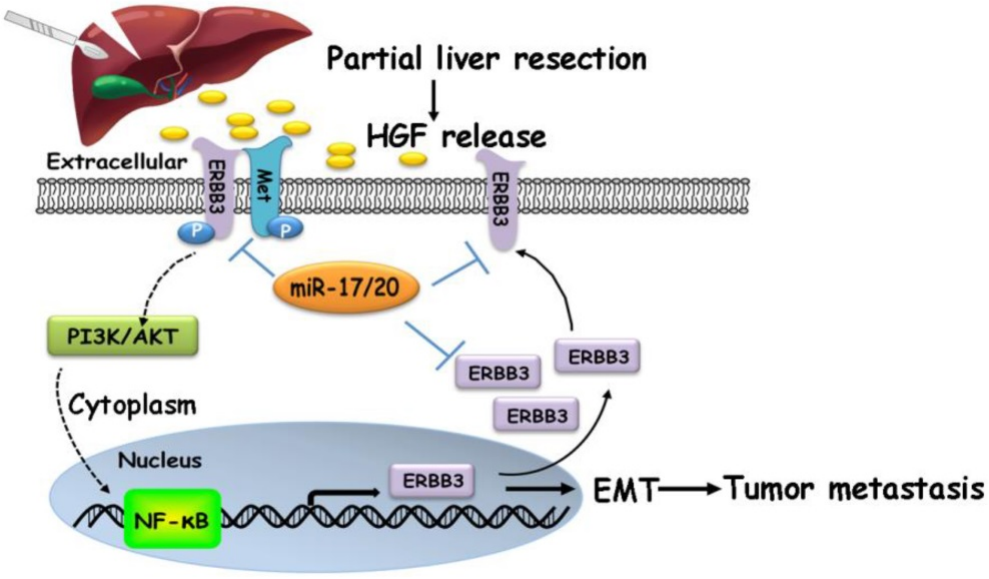
A proposed model illustrating the miR-17-5p and miR-20a-5p suppressive role on HCC metastasis by blocking HGF/ERBB3-NF-κB positive feedback loop after hepatectomy.

Our findings suggested that HGF/ERBB3 and NF-κB form a positive feedback loop and that ERBB3 promotes HCC cells' sensitivity to HGF stimuli. We finally sought to determine whether inhibition of ERBB3 by miR-17-5p and miR-20a-5p is an effective strategy of suppressing HCC metastasis after hepatectomy. Our results indicated that HGF-induced ERBB3, p-ERBB3, p-AKT and NF-κB expressions were significantly inhibited in miR-17-5p or miR-20a-5p mimics transfected HCCLM3 cells (Figure 8A). Moreover, HGF-induced Vimentin as well as Twist and Snail, two key regulation factors of EMT, were significantly decreased, while E-cadherin markedly increased in miR-17-5p or miR-20a-5p mimics transfected cells (Figure 8B). Using tail vein injection xenograft model, the responses of HCCLM3-miR*^NC^*, HCCLM3-miR-17-5p*^OE^* or HCCLM3-miR-20a-5p*^OE^* cells on partial mouse liver resection (Figure S4D) were subsequently gauged since the second post-operative day. Six weeks after injection, tumor foci in the lung of recipient were reduced by approximately 4 folds in HCCLM3-miR-17-5p*^OE^* xenograft and 3.9 folds in HCCLM3-miR-20a-5p*^OE^* xenograft compared with HCCLM3-miR*^NC^* xenograft (Figure 8C-D). Taken together, our observations indicate that miR-17-5p and miR-20a-5p play a suppressive role on HCC metastasis by blocking HGF/ERBB3-NF-κB positive feedback loop after hepatectomy (Scheme 1).

## Discussion

It is well known that surgical resection is the first-line choice for HCC patients at early stage. After partial hepatectomy, hepatic growth and liver regeneration are immediately provoked as a compensative process for sudden liver dysfunction, accompanied with a tremendous change of liver microenvironment and an outburst of a wide spectrum of cytokines and growth factors. Of which, the functions of HGF were controversial. Some research regarded HGF as an indispensable factor for liver regeneration because of its rapid and sustained signal during microenvironment remodeling postoperation 35-38. While others expounded HGF was usually negatively correlated with patient survival and deemed a poor biomarker in tumor development, metastasis and recurrence 28. Herein, by *in vitro* and *in vivo* model, HGF was confirmed to promote HCC metastasis. Although exposed in a similar context, no tumor recurrence and metastasis occurred in about 40% HCC patients for more than 5 years after tumor resection. The phenomena remind us that a differential response of residual tumor cells existed on the sudden tremendous environmental programming. That is to say, heterogeneous HCC populations might respond in disparity on HGF-stimulated tumor growth and metastasis. However, the underlying mechanisms are still unclear and need be elucidated.

Mounting evidences have emerged that miRNAs are the major drivers on HCC metastasis at the post-transcriptional level 39-41. Therefore, identifycations of metastasis-related miRNAs and their direct target genes are critical steps for understanding miRNA mechanisms on HCC metastatic progression. In the present study, miR-17-92 cluster was found down-regulated obviously in high metastatic HCC cell lines using genome-wide miRNA analysis. As reported miR-17-5p or miR-20a-5p, two abundant members of miR-17-92 family, individually functioned as antimiRs in HCC migration, invasion and metastasis using gain- or loss-of-function strategies 42,43. However, no additive or synergistic effects of miR-17-5p and miR-20a-5p on HCC metastasis exhibited after co-transfections (unpublished data). When metastasis-related differentially expressed mRNAs were overlapped with the predicted genes of miR-17-92 cluster, ERBB3 was a common downstream target of miR-17-5p and miR-20a-5p with the same seed sequence at 3'UTR region. However, the redundantly regulatory mechanism on ERBB3 expression might ensure the biological importance of miR-17-92 cluster on HCC metastasis.

ERBB3 is an essential member of EGFR family with receptor tyrosine kinase activity and serves as an oncogene in cancer development and progression 30,31,44. Overexpression of ERBB3 in lung cancers usually correlates with poor survivals and high brain metastases 32. Also, ERBB3 is often highly expressed in melanomas and even more highly expressed in its metastatic foci 45. Knockdown of ERBB3 in melanoma can reduce tumor cell migration and invasion 46. However, its biological significances on HCC metastasis and progression are not yet determined. ERBB3 signaling was associated with HCC EMT, migration and invasion by either inhibition of miR-296-5p in our previous work 47, or blockade of miR-17-5p/miR-20a-5p in the present work by *in vitro* and *in vivo* assay. More importantly, the levels of ERBB3 were inversely correlated with metastasis-free survival and overall survival in postoperative HCC patients, which was also be validated by TCGA database.

As metastasis is a low efficient event during the tumor invasive-metastatic process, only a small subset, which is adapted to the programming of tumor environments, will successfully evolve from the large heterogeneous populations and finally complete a metastatic growth in the second organs. In the light of this hypothesis, a small subset expressed with lower endogenous levels of miR-17-92 cluster was indeed found in HCC with high metastatic potentials and exhibited more sensitive on HGF stimuli after hepatectomy via HGF/ERBB3-NF-κB positive feedback loop. If attenuated this feedback loop by miR-17-5p and miR-20a-5p, HCC metastasis might be largely suppressed in postoperation.

In conclusion, a novel insight of miR-17-5p and miR-20a-5p on HCC metastasis was identified in the study, and the miRNAs might function as antimiRs against ERBB3 in HCC metastasis after partial liver resection.

## Supplementary Material

Supplementary figures and tables.

## Abbreviations

HCC: hepatocellular carcinoma
miRNA: microRNA
EMT: epithelial-mesenchymal transition
NC: negative control
WT: none vectors
FDR: false discovery rate
RPKM: Reads Per Kb per Million reads
DEGs: differentially expressed genes
TMA: thick tissue microarray
FFPE: Formalin-Fixed and Paraffin-Embedded
FISH: fluorescence *in situ* hybridization
OS: overall survival
PFS: progression free survival
ELISA: Enzyme-linked immunosorbent assay
PLA: proximity ligation assay
ChIP: Chromatin immunoprecipitation
CoIP: Co-immunoprecipitation
HGF: hepatocyte growth factor
EGF: epidermal growth factor
AFP: alpha-fetoprotein
GGT: gamma glutamyl transferase
TNM: tumor node-metastasis
NA: not available
