# Supplementary material for: miR-17-5p and miR-20a-5p suppress postoperative metastasis of hepatocellular carcinoma via blocking HGF/ERBB3-NF-κB positive feedback loop
Source: Theranostics. 2020 Feb 19;10(8):3668–83. doi: 10.7150/thno.41365 (PMC7069088; doi:10.7150/thno.41365)
Supplement: Supplementary file 1 — Supplementary figures and tables. [file thnov10p3668s1.pdf]

Supplementary Figure S1. HGF enhanced HCC metastasis.

(A) Serum EGF level in HCC patients pre- and post-operation. Student's t-test. **(B&D)** Representative images of motility assays. Bars, 200 μm. (**C&E)** Motility assays of HCCLM3 and HepG2 cells with indicated treatment. n=3. Student's t-test. **p<0.01. Data are mean ± SD and are representative of three independent experiments.

Supplementary Figure S2. The suppressive effects of miR-17-5p and miR-20a-5p on HCC metastasis *in vitro.*

(A) Representative images of HCCLM3 cells transfected with miR-17-5p and miR-20a-5p mimics overexpression lentiviral vectors, respectively. HepG2 cells was transfected with miR-17-5p and miR-20a-5p knockdown lentiviral vectors. Bars, 200 μm. **(B&C)** Representative images of invasion assays. Bars, 200 μm. **(D&E)** Invasion assays of HCCLM3 and HepG2 cells with indicated treatment. n=3. Student's t-test. ***p<0.001. Data are mean ± SD and are representative of three independent experiments.

Supplementary Figure S3. ERBB3 is a common downstream target of miR-17-5p and miR-20a-5p.

(A&B) The mRNA level of ERBB3 detected by RT-PCR**.** Student's t-test. **(C)** Luciferase activity was assayed in HEK-293T cells. Student's t-test.*p<0.05; **p<0.01; ***p<0.001. **(D)** Representative images of HepG2 migration after ERBB3 modulations. Bars, 200μm. **(E)** Statistical results of HepG2 motilities after indicated treatments. n=3. Student's t-test.

*p<0.05; **p<0.01. **(F)** Representative wound-healing images of HCCLM3 cells after ERBB3 modulations. Magnification 40×. Data are mean ± SD and are representative of three independent experiments.

Supplementary Figure S4. ERBB3 levels directly induced by HGF.

(A) AKT signaling in HCCLM3 cells treated with HGF. **(B)** Diagrammatic illustration of the interaction of ERBB3 and MET. **(C)** ERBB3 immunoprecipitation was performed and all samples were immunobloted using the indicated antibodies. **(D)** Representative images of partial liver of nude mice after liver resection. **(E&F)** Kaplan-Meier analyses of OS and RFS (recurrence free survival) for ERBB3 in human HCCs by TCGA dataset using SPSS 22.0 software. (n=370, p=0.077; n=316, p=0.011, respectively). （**G&H**） Kaplan-Meier analyses of OS for miR-17-5p and miR-20a-5p in human HCCs by TCGA dataset using SPSS 22.0 software. (n=252, p=0.023; n=252, p=0.027, respectively).

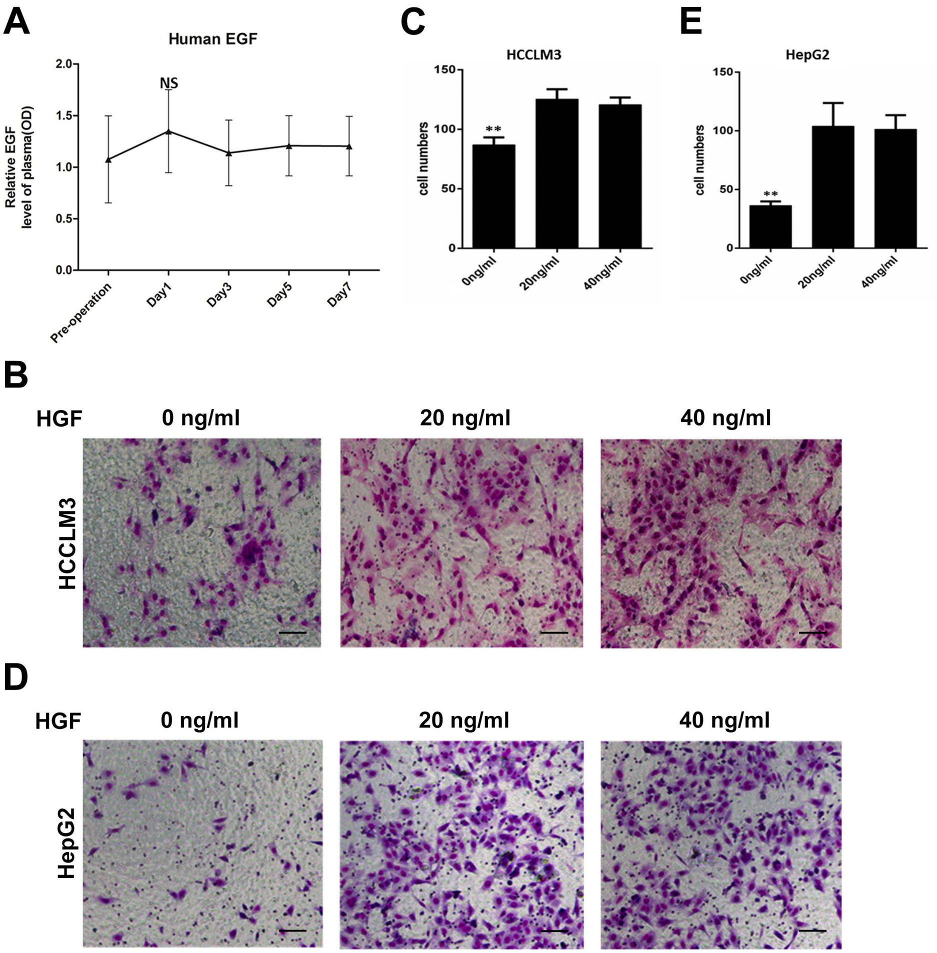
A
Human EGF
Relative EGF level of plasma(OD)
NS
Pre-operation
Day1
Day3
Day5
Day7
C
HCCLM3
cell numbers
**
0ng/ml
20ng/ml
40ng/ml
E
HepG2
cell numbers
**
0ng/ml
20ng/ml
40ng/ml
B
HGF
0 ng/ml
20 ng/ml
40 ng/ml
HCCLM3
D
HGF
0 ng/ml
20 ng/ml
40 ng/ml
HepG2

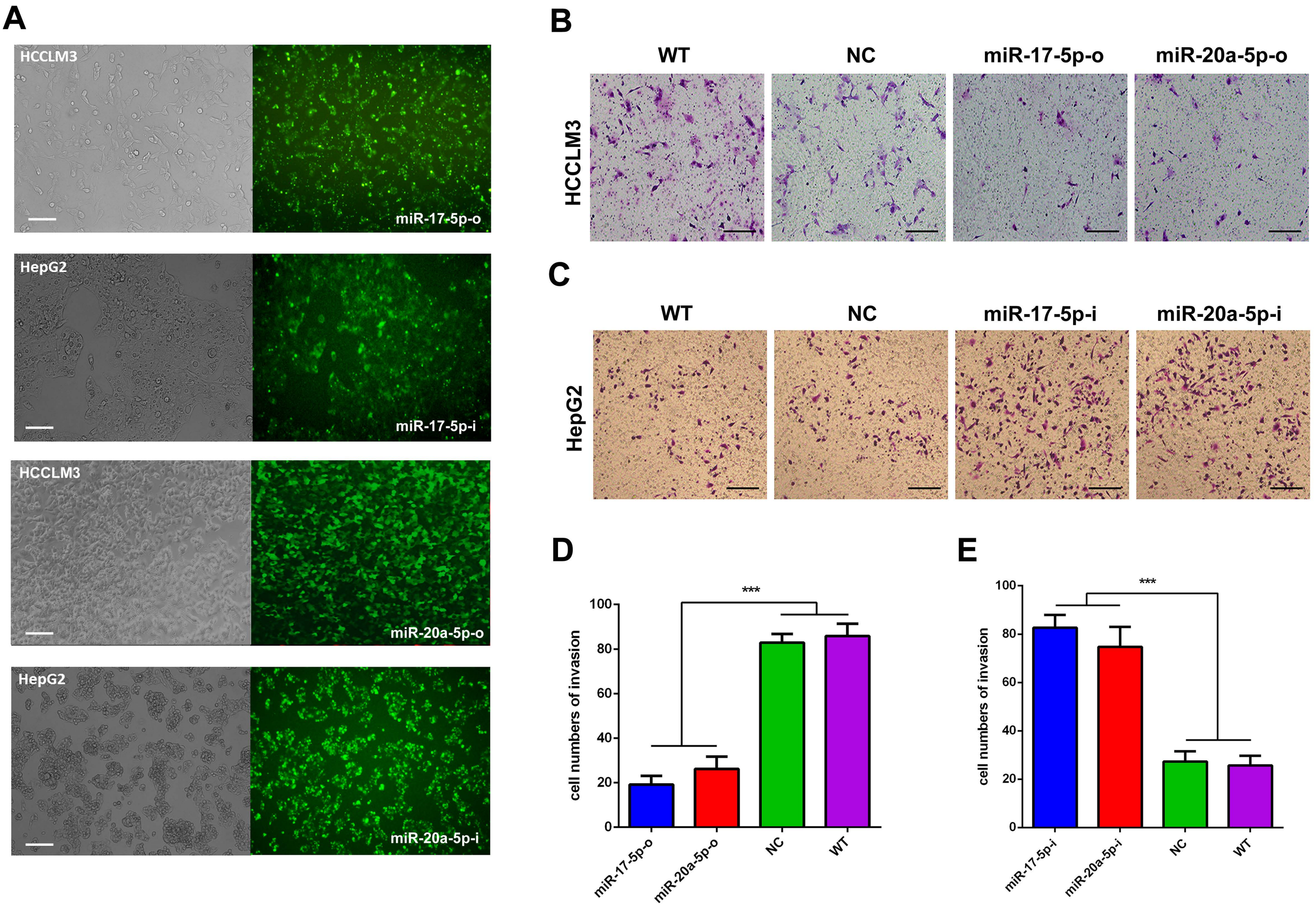
A
HCCLM3
miR-17-5p-o
HepG2
miR-17-5p-i
HCCLM3
miR-20a-5p-o
HepG2
miR-20a-5p-i
B
WT
NC
miR-17-5p-o
miR-20a-5p-o
HCCLM3
C
WT
NC
miR-17-5p-i
miR-20a-5p-i
HepG2
D
cell numbers of invasion
***
0
20
40
60
80
100
miR-17-5p-o
miR-20a-5p-o
NC
WT
E
cell numbers of invasion
***
0
20
40
60
80
100
miR-17-5p-i
miR-20a-5p-i
NC
WT

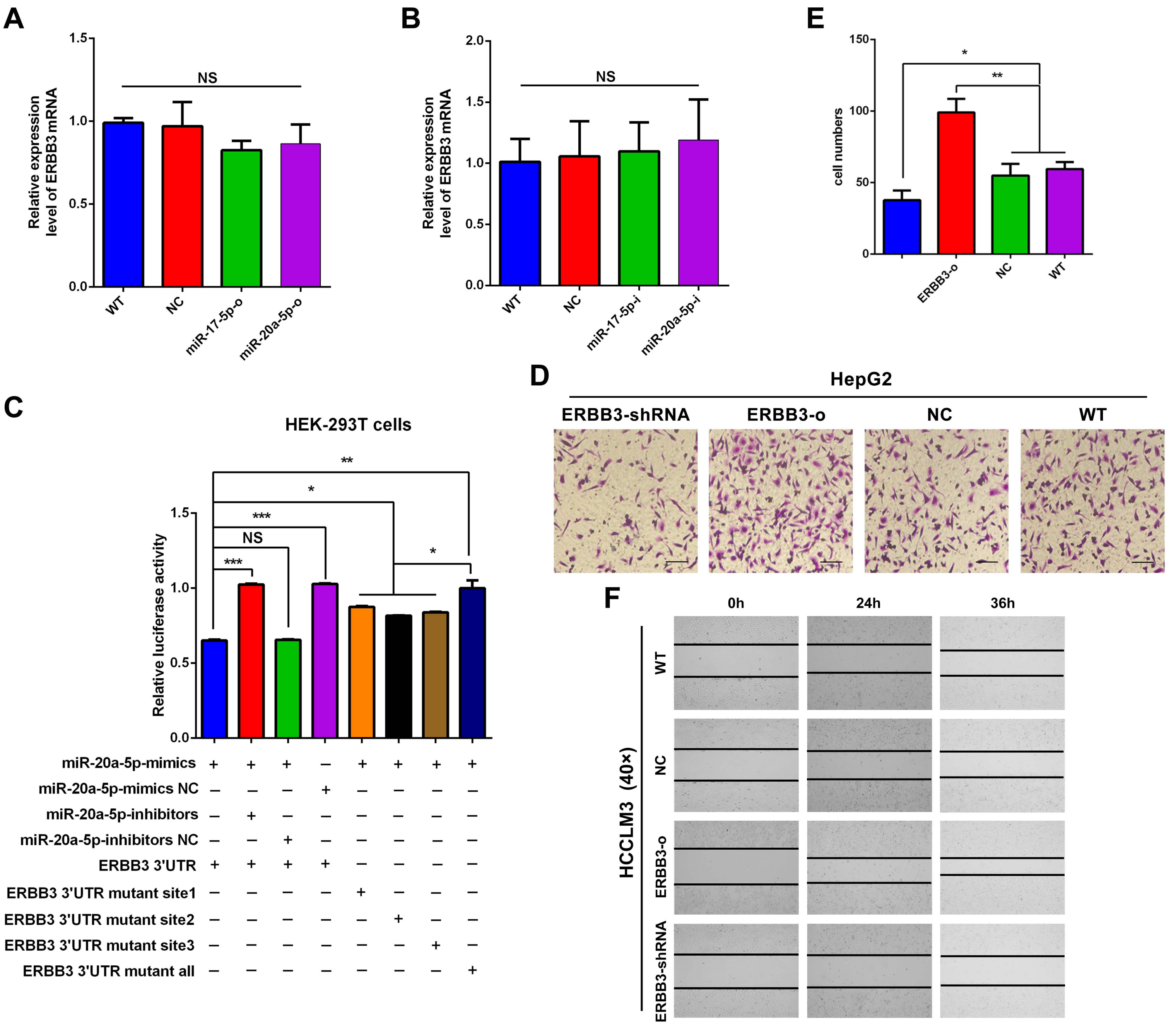
A
Relative expression level of ERBB3 mRNA
NS
WT
NC
miR-17-5p-o
miR-20a-5p-o
B
Relative expression level of ERBB3 mRNA
NS
WT
NC
miR-17-5p-i
miR-20a-5p-i
E
cell numbers
ERBB3-o
NC
WT
C
HEK-293T cells
Relative luciferase activity
miR-20a-5p-mimics
miR-20a-5p-mimics NC
miR-20a-5p-inhibitors
miR-20a-5p-inhibitors NC
ERBB3 3'UTR
ERBB3 3'UTR mutant site1
ERBB3 3'UTR mutant site2
ERBB3 3'UTR mutant site3
ERBB3 3'UTR mutant all
D
HepG2
ERBB3-shRNA
ERBB3-o
NC
WT
F
0h
24h
36h
HCCLM3 (40×)
WT
NC
ERBB3-o
ERBB3-shRNA

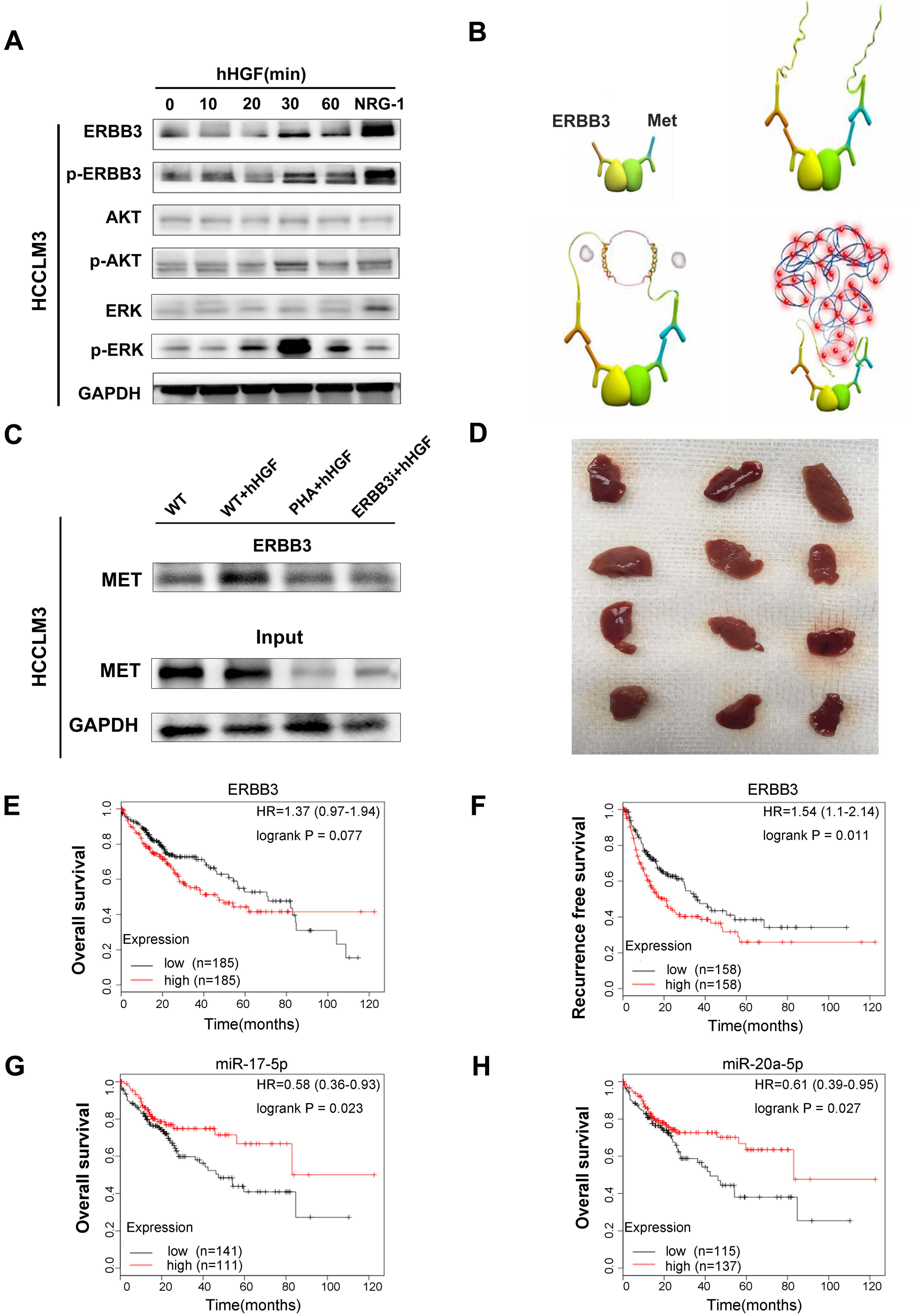
A
hHGF(min)
0
10
20
30
60
NRG-1
ERBB3
p-ERBB3
AKT
p-AKT
ERK
p-ERK
GAPDH
HCCLM3
B
ERBB3
Met
C
WT
WT+hHGF
PHA+hHGF
ERBB3i+hHGF
ERBB3
MET
Input
MET
GAPDH
HCCLM3
D
E
ERBB3
HR=1.37 (0.97-1.94)
logrank P = 0.077
Overall survival
Expression
low (n=185)
high (n=185)
Time(months)
F
ERBB3
HR=1.54 (1.1-2.14)
logrank P = 0.011
Recurrence free survival
Expression
low (n=158)
high (n=158)
Time(months)
G
miR-17-5p
HR=0.58 (0.36-0.93)
logrank P = 0.023
Overall survival
Expression
low (n=141)
high (n=111)
Time(months)
H
miR-20a-5p
HR=0.61 (0.39-0.95)
logrank P = 0.027
Overall survival
Expression
low (n=115)
high (n=137)
Time(months)

Supplementary Table S1

miR-name	1-Expression	2-Expression	3-Expression	log2 Ratio(2/1)	log2 Ratio(3/1)	P-value(2/1)	P-value(3/1)
hsa-let-7a-3p	5.9111	1.8945	2.601	-1.64160949	-1.18436023	3.17E-05	0.00045289
hsa-miR-106b-5p	223.6019	86.6034	71.637	-1.36843688	-1.64215562	2.12E-114	8.11E-171
hsa-miR-10a-5p	143.1908	296.3462	344.4214	1.04934476	1.26623598	1.24E-103	9.48E-180
hsa-miR-1224-5p	1.1211	0.01	0.1084	-6.80877116	-3.37047831	0.00237241	0.00478519
hsa-miR-122-5p	5.4015	2.571	1.9508	-1.07103049	-1.46929422	0.004109806	7.72E-05
hsa-miR-1252	1.4268	0.01	0.3251	-7.15663931	-2.13382766	0.000440281	0.010941928
hsa-miR-1255a	27.1094	11.908	11.3796	-1.18686206	-1.25234334	1.53E-12	2.73E-15
hsa-miR-1255b-5p	3.567	1.7591	0.7586	-1.01987372	-2.23329994	0.026324477	2.17E-05
hsa-miR-125a-5p	286.9931	133.4234	125.9338	-1.10500434	-1.1883505	2.04E-105	7.14E-135
hsa-miR-1262	8.1532	2.7064	1.5173	-1.59099132	-2.42586004	1.59E-06	9.42E-12
hsa-miR-1277-5p	4.6881	1.7591	1.4089	-1.41416585	-1.73443413	0.000926808	3.43E-05
hsa-miR-128	1566.844	771.0413	596.7225	-1.0229815	-1.39272947	0	0
hsa-miR-1292	9.4781	4.7361	4.3351	-1.00089834	-1.12853261	0.00029093	1.81E-05
hsa-miR-1293	55.5438	21.3802	20.2665	-1.37735053	-1.45452892	5.76E-30	6.19E-37
hsa-miR-139-5p	7.6436	3.7889	1.4089	-1.01247322	-2.43968307	0.001049056	3.67E-11
hsa-miR-146b-5p	79.6977	35.8592	21.3502	-1.15219479	-1.90028851	1.68E-32	1.34E-75
hsa-miR-149-3p	6.3187	2.977	2.1675	-1.08576855	-1.54359578	0.001655714	9.00E-06
hsa-miR-149-5p	2.446	0.5413	0.6503	-2.17592411	-1.91124707	0.001606845	0.001577607
hsa-miR-151b	17.1218	6.6306	7.0445	-1.36862305	-1.28126516	3.82E-10	1.70E-10
hsa-miR-15a-5p	23.9501	11.2314	10.4042	-1.09249391	-1.20286564	4.96E-10	6.27E-13
hsa-miR-15b-5p	103.6477	51.5561	47.6858	-1.00747306	-1.12005648	5.55E-34	1.55E-45
hsa-miR-16-2-3p	20.6888	7.0365	9.7539	-1.55592006	-1.08479888	3.64E-14	7.56E-10
hsa-miR-17-3p	19.262	9.3369	6.8277	-1.04474197	-1.49628593	7.63E-08	2.51E-14
hsa-miR-17-5p	53.5055	15.8322	14.1974	-1.75682546	-1.91406045	2.21E-40	8.41E-52
hsa-miR-181a-2-3p	53.1997	20.839	13.6555	-1.35213206	-1.96193597	5.07E-28	3.53E-53
hsa-miR-181b-3p	2.344	0.9472	0.7586	-1.30723158	-1.62756129	0.030371568	0.005647231
hsa-miR-186-5p	31.3899	14.2084	15.0644	-1.14355633	-1.05915722	1.44E-13	1.00E-13
hsa-miR-18a-5p	3.0575	0.5413	0.3251	-2.49785221	-3.23339704	0.000117096	1.79E-06
hsa-miR-1908	1.6306	0.2706	0.4335	-2.59116918	-1.91129902	0.005234325	0.010790528
hsa-miR-191-5p	3787.9834	1648.0366	1804.5815	-1.20068173	-1.06976571	0	0
hsa-miR-199a-3p	3.1594	0.8119	0.9754	-1.96027665	-1.69558473	0.000709539	0.000875303
hsa-miR-199b-3p	3.1594	0.8119	0.9754	-1.96027665	-1.69558473	0.000709539	0.000875303
hsa-miR-19a-3p	1.6306	0.406	0.1084	-2.00585129	-3.91096626	0.01623969	0.000235818

hsa-miR-19b-3p	28.7401	9.4723	4.877	-1.6012784	-2.5589992	7.29E-20	3.27E-40
hsa-miR-20a-5p	38.2182	8.7957	6.3942	-2.11938953	-2.57942406	5.03E-37	2.05E-53
hsa-miR-219-5p	1.3249	0.01	0.01	-7.04973965	-7.04973965	0.00077188	0.000186475
hsa-miR-221-3p	25543.3073	11203.9151	8926.2355	-1.18894238	-1.51682156	0	0
hsa-miR-221-5p	2219.8145	810.4188	784.5394	-1.45369957	-1.50052131	0	0
hsa-miR-222-3p	83827.7833	33203.356	26612.8901	-1.33609941	-1.65530329	0	0
hsa-miR-222-5p	9.9877	2.4357	1.3005	-2.03581604	-2.94108609	3.14E-10	4.78E-17
hsa-miR-2355-3p	3.3632	0.6766	0.6503	-2.31345949	-2.37065725	0.000105532	1.96E-05
hsa-miR-25-5p	1315.3173	516.779	645.1669	-1.34779152	-1.02766654	0	0
hsa-miR-26a-5p	363.2257	163.1934	148.0427	-1.15428357	-1.29485293	2.44E-142	2.19E-194
hsa-miR-29a-5p	4.3824	2.1651	1.5173	-1.01728751	-1.53021481	0.013554485	0.000252365
hsa-miR-301a-3p	2.6498	0.6766	0.5419	-1.96950839	-2.28978492	0.001980279	0.000221754
hsa-miR-30c-1-3p	6.6245	3.2476	2.8178	-1.02843762	-1.23324235	0.002040201	0.0001247
hsa-miR-30e-3p	15.4911	7.7131	5.0937	-1.00605687	-1.60465369	3.02E-06	6.58E-13
hsa-miR-3121-3p	4.0766	1.6238	1.6257	-1.32799246	-1.32630535	0.00331556	0.001531964
hsa-miR-3138	5.6053	2.4357	1.734	-1.20245514	-1.69268769	0.001336639	8.11E-06
hsa-miR-3140-3p	2.9555	0.5413	0.5419	-2.44890193	-2.44730367	0.000182868	4.80E-05
hsa-miR-3144-5p	12.4337	3.3829	4.6602	-1.87792324	-1.4157919	2.53E-11	4.45E-09
hsa-miR-3153	1.7326	0.2706	0.6503	-2.67870488	-1.41376129	0.003292202	0.033173034
hsa-miR-3164	1.5287	0.2706	0.3251	-2.49807157	-2.23334985	0.008280761	0.00661591
hsa-miR-3165	3.7709	1.7591	0.867	-1.10007139	-2.12080499	0.015004062	2.31E-05
hsa-miR-3171	5.4015	1.2179	0.867	-2.14896442	-2.6392562	2.04E-06	6.90E-09
hsa-miR-3173-5p	1.1211	0.2706	0.1084	-2.05068122	-3.37047831	0.048754295	0.00478519
hsa-miR-3179	5.6053	1.4885	2.0592	-1.91293237	-1.44470763	6.97E-06	6.95E-05
hsa-miR-335-5p	11.2107	4.4655	4.6602	-1.32798274	-1.26641259	7.88E-07	3.18E-07
hsa-miR-34a-5p	25.9884	7.9838	5.2021	-1.70272033	-2.32070178	1.34E-19	9.53E-33
hsa-miR-3605-5p	5.0958	2.1651	2.3843	-1.234875	-1.09574288	0.001820383	0.002244828
hsa-miR-362-3p	4.9938	2.0298	0.3251	-1.29880046	-3.94118258	0.001333897	2.29E-11
hsa-miR-365a-5p	128.515	61.4343	55.814	-1.06482049	-1.20323781	1.67E-45	1.32E-62
hsa-miR-3691-5p	1.8345	0.6766	0.5419	-1.43901182	-1.75928835	0.041536034	0.010232506
hsa-miR-374a-3p	90.093	35.318	37.6067	-1.35101137	-1.2604253	2.82E-46	1.23E-47
hsa-miR-374a-5p	64.4104	29.7699	21.567	-1.11343928	-1.57846814	2.99E-25	8.62E-48
hsa-miR-375	2.0383	0.5413	0.5419	-1.91286611	-1.91126785	0.008415903	0.004093892
hsa-miR-3909	3.2613	0.8119	0.9754	-2.00607321	-1.74138128	0.00047444	0.000568175
hsa-miR-3916	3.3632	1.6238	1.0838	-1.05046062	-1.63373602	0.027359807	0.000813152

hsa-miR-425-5p	29.3516	11.7727	13.6555	-1.31799392	-1.10395701	1.42E-15	1.01E-13
hsa-miR-4428	1.7326	0.2706	0.2168	-2.67870488	-2.99850196	0.003292202	0.000656234
hsa-miR-4436b-3p	6.1149	1.8945	1.3005	-1.69051177	-2.23326251	1.54E-05	2.12E-08
hsa-miR-4440	1.1211	0.1353	0.01	-3.05068122	-6.80877116	0.014602643	0.00070209
hsa-miR-4454	0.5096	6.4953	7.0445	3.67195898	3.78906013	4.32E-13	1.76E-15
hsa-miR-4467	13.1471	5.2774	5.3105	-1.31684536	-1.307825	1.04E-07	1.35E-08
hsa-miR-448	4.4843	0.6766	1.1921	-2.72850772	-1.91137754	7.69E-07	1.52E-05
hsa-miR-4488	0.3057	4.7361	4.3351	3.95351109	3.82587682	2.23E-10	4.91E-10
hsa-miR-4508	0.1019	3.7889	2.3843	5.21655311	4.54833981	8.02E-10	1.49E-06
hsa-miR-4521	137.6874	37.0771	28.178	-1.89279623	-2.28875542	8.54E-112	1.31E-163
hsa-miR-452-3p	4.1785	2.0298	1.3005	-1.04164755	-1.68391874	0.014070252	0.000130592
hsa-miR-454-3p	2.0383	0.6766	0.5419	-1.59099132	-1.91126785	0.020204653	0.004093892
hsa-miR-455-3p	20.5869	10.0135	8.7785	-1.03978028	-1.22968025	3.07E-08	1.16E-11
hsa-miR-455-5p	25.5807	12.3139	12.0298	-1.05476799	-1.08844309	4.09E-10	6.65E-12
hsa-miR-486-3p	2.8536	0.8119	0.9754	-1.81340917	-1.54871724	0.002311276	0.003083896
hsa-miR-5001-5p	1.4268	0.406	0.1084	-1.81323149	-3.71834646	0.036765411	0.000794722
hsa-miR-505-5p	43.314	13.8024	17.557	-1.64991426	-1.30278706	2.28E-30	6.48E-25
hsa-miR-548h-5p	8.0513	3.7889	1.6257	-1.08744268	-2.30816069	0.000361822	4.87E-11
hsa-miR-548j	4.8919	1.6238	2.0592	-1.59102096	-1.24831095	0.000223847	0.000902674
hsa-miR-548t-5p	6.3187	2.0298	1.9508	-1.63829019	-1.69556189	1.71E-05	2.04E-06
hsa-miR-549	2.6498	0.2706	0.5419	-3.29164973	-2.28978492	4.30E-05	0.000221754
hsa-miR-5584-5p	1.223	0.2706	0.3251	-2.17619066	-1.91146894	0.031637511	0.029114891
hsa-miR-582-5p	3.567	1.4885	1.5173	-1.26085199	-1.23320485	0.008499231	0.005168103
hsa-miR-584-5p	8.357	2.4357	2.601	-1.77864869	-1.68391874	1.49E-07	5.12E-08
hsa-miR-590-3p	6.4207	1.7591	0.6503	-1.86789309	-3.30355326	2.09E-06	1.41E-12
hsa-miR-590-5p	3.9747	1.2179	1.3005	-1.70645029	-1.61177957	0.000500934	0.000301348
hsa-miR-625-3p	23.5424	4.1949	9.3204	-2.48855308	-1.33679762	5.59E-28	9.64E-15
hsa-miR-652-3p	5.4015	1.7591	2.4927	-1.6185226	-1.11565084	8.41E-05	0.001403399
hsa-miR-671-5p	1.223	0.01	0.01	-6.93428059	-6.93428059	0.001353224	0.000361831
hsa-miR-675-5p	4.5862	13.2612	16.1481	1.53184015	1.81599325	7.50E-10	7.56E-16
hsa-miR-676-3p	3.3632	0.5413	0.2168	-2.63533428	-3.95539791	3.02E-05	5.12E-08
hsa-miR-7-1-3p	4.0766	1.2179	1.6257	-1.74297072	-1.32630535	0.000345494	0.001531964
hsa-miR-7-5p	360.8817	135.9945	141.8652	-1.40797768	-1.34700525	2.18E-191	3.98E-205
hsa-miR-769-3p	2.446	0.8119	0.9754	-1.59105045	-1.32635853	0.010387645	0.014965623
hsa-miR-92a-1-5p	206.684	40.866	48.6612	-2.33845377	-2.08658291	3.68E-217	4.12E-218

hsa-miR-92a-3p	1035.9678	500.6762	478.7002	-1.04902938	-1.11378485	0	0
hsa-miR-93-5p	909.593	396.0755	425.5956	-1.19944569	-1.09573792	0	0
hsa-miR-942	3.0575	1.3532	0.9754	-1.17597742	-1.64828662	0.021603258	0.001340431
hsa-miR-99b-5p	622.2941	222.192	237.7786	-1.48578969	-1.38797769	0	0

Supplementary Table S2

GeneID	Gene_leng	1-Express	2-Express	3-Express	1-RPKM	2-RPKM	3-RPKM	log2 Rati	log2 Rati	Up-Down-F	P-value(2	P-value(3	FDR(2/1)	FDR(3/1)	Symbol
121355	822	0	140	75	0.001	36.9474	19.30963	15.17319	14.23703	Up	0	0	0	0	GTSF1
3957	543	0	17	14	0.001	6.791674	5.45648	12.72955	12.41375	Up	9.68E-06	8.92E-05	9.84E-05	0.000677	LGALS2
6288	678	0	16	16	0.001	5.119387	4.994301	12.32176	12.28607	Up	1.91E-05	2.35E-05	0.000183	0.000204	SAA1
23532	2776	0	83	37	0.001	6.48614	2.820763	12.66314	11.46187	Up	0	1.90E-11	0	4.10E-10	PRAME
139420	3133	0	30	37	0.001	2.077249	2.499342	11.02046	11.28733	Up	1.40E-09	1.90E-11	2.64E-08	4.10E-10	SMEK3P
57507	5645	0	43	27	0.001	1.652465	1.012242	10.6904	9.983338	Up	2.03E-13	1.51E-08	7.98E-12	2.26E-07	ZNF608
93986	6448	0	24	29	0.001	0.807447	0.951826	9.657223	9.894553	Up	8.29E-08	3.98E-09	1.20E-06	6.46E-08	FOXP2
57458	5867	0	18	21	0.001	0.665555	0.757509	9.378415	9.565119	Up	4.90E-06	8.32E-07	5.22E-05	9.42E-06	TMCC3
405753	1451	9	229	459	1.381791	34.23701	66.94678	4.630945	5.598403	Up	2.59E-06	2.31E-06	2.93E-05	2.44E-05	DUOXA2
213	2264	25	517	736	2.459976	49.53838	68.79958	4.331831	4.805683	Up	9.37E-11	6.88E-11	2.04E-09	1.38E-09	ALB
50506	6428	58	837	1654	2.010108	28.24732	54.45579	3.812769	4.75974	Up	9.77E-15	0	5.09E-13	0	DUOX2
54866	880	4	34	100	1.012616	8.381543	24.04926	3.049129	4.569834	Up	4.40E-05	4.14E-05	0.000392	0.000339	PPP1R14D
7104	1624	14	345	303	1.920478	46.08513	39.48581	4.584764	4.361797	Up	1.16E-07	9.76E-08	1.65E-06	1.28E-06	TM4SF4
56034	3079	2	52	43	0.144706	3.663712	2.955583	4.662106	4.352242	Up	0.000113	0.00011	0.000904	0.000818	PDGFC
5284	4295	6	73	125	0.311211	3.687121	6.159298	3.566528	4.3068	Up	1.49E-05	1.38E-05	0.000146	0.000127	PIGR
7113	3250	8	34	160	0.54837	2.269464	10.41888	2.049129	4.247906	Up	6.49E-05	4.26E-06	0.000557	4.29E-05	TMPRSS2
3822	1223	3	48	59	0.546465	8.514173	10.20963	3.961666	4.223658	Up	7.07E-05	6.81E-05	0.0006	0.000531	KLRC2
84830	1866	5	113	98	0.596933	13.13695	11.11473	4.459917	4.218759	Up	2.58E-05	2.43E-05	0.000241	0.00021	C6orf105
57211	6980	14	264	274	0.446828	8.204955	8.307676	4.198705	4.216655	Up	1.16E-07	9.76E-08	1.65E-06	1.28E-06	GPR126
8470	6344	5	77	95	0.17558	2.633027	3.169165	3.906525	4.173905	Up	2.58E-05	2.43E-05	0.000241	0.00021	SORBS2
9076	3452	12	93	207	0.774422	5.844399	12.69065	2.915863	4.034502	Up	4.10E-07	3.53E-07	5.31E-06	4.27E-06	CLDN1
81563	10308	2	46	32	0.043224	0.96808	0.656992	4.485228	3.925978	Up	0.000113	0.00011	0.000904	0.000818	C1orf21
3934	840	141	1041	2154	37.39445	268.8433	542.6887	2.845869	3.859229	Up	6.88E-15	0	3.67E-13	0	LCN2
338	14121	19	80	278	0.299747	1.229001	4.166427	2.035667	3.796991	Up	5.29E-09	3.69E-09	9.15E-08	6.02E-08	APOB
6590	598	6	37	83	2.235205	13.42234	29.37388	2.586157	3.716055	Up	1.64E-05	1.38E-05	0.000159	0.000127	SLPI
8875	2034	5	53	69	0.547629	5.652657	7.179307	3.367659	3.712574	Up	2.58E-05	2.43E-05	0.000241	0.00021	VNN2
7412	3220	34	231	447	2.352287	15.56266	29.37894	2.725952	3.642646	Up	4.33E-13	2.90E-13	1.52E-11	8.97E-12	VCAM1
718	5101	21	207	267	0.917131	8.803244	11.07746	3.262836	3.594356	Up	1.27E-09	9.79E-10	2.41E-08	1.71E-08	C3
2919	1119	9	113	114	1.79176	21.90666	21.56052	3.61192	3.588943	Up	2.59E-06	2.31E-06	2.94E-05	2.44E-05	CXCL1
25890	4488	2	31	25	0.099276	1.49843	1.178885	3.915863	3.569834	Up	0.000113	0.000115	0.000904	0.000854	ABI3BP
119467	1174	4	30	48	0.75903	5.543459	8.652817	2.868557	3.51094	Up	4.85E-05	4.14E-05	0.000428	0.000339	CLRN3
10404	1928	4	65	47	0.46219	7.313648	5.159115	3.984034	3.480567	Up	4.35E-05	4.14E-05	0.00039	0.000339	PGCP
6512	2677	10	86	113	0.832183	6.969118	8.933353	3.066003	3.424229	Up	1.41E-06	1.25E-06	1.67E-05	1.38E-05	SLC1A7
183	2587	20	233	220	1.722269	19.53832	17.99744	3.503924	3.385409	Up	2.44E-09	1.90E-09	4.40E-08	3.20E-08	AGT
687	5208	4	44	43	0.171102	1.832776	1.747358	3.421098	3.352242	Up	4.35E-05	4.14E-05	0.000389	0.000339	KLF9

25837	1641	4	32	40	0.543024	4.23028	5.158647	2.961666	3.247906	Up	4.51E-05	4.14E-05	0.000401	0.000339	RAB26
55304	3855	7	33	68	0.404521	1.857023	3.733094	2.198705	3.206086	Up	4.48E-05	7.74E-06	0.000399	7.45E-05	SPTLC3
1.01E+08	4773	16	130	153	0.746785	5.908533	6.783977	2.984034	3.183366	Up	3.25E-08	2.65E-08	5.05E-07	3.82E-07	DCDC5
10537	1006	52	227	491	11.51523	48.95033	103.2923	2.087775	3.165117	Up	5.95E-14	9.77E-15	2.73E-12	3.81E-13	UBD
23150	5261	20	119	170	0.846894	4.906891	6.838566	2.534556	3.013441	Up	2.44E-09	1.90E-09	4.39E-08	3.19E-08	FRMD4B
9947	4337	43	323	362	2.208749	16.15626	17.66459	2.870792	2.999559	Up	1.25E-13	8.55E-14	5.23E-12	3.00E-12	MAGEC1
79365	3796	6	91	50	0.352121	5.200474	2.787586	3.884498	2.984871	Up	1.49E-05	1.38E-05	0.000146	0.000127	BHLHE41
10628	2953	26	116	213	1.96145	8.521622	15.26513	2.119208	2.960248	Up	4.90E-11	3.56E-11	1.11E-09	7.36E-10	TXNIP
5742	5093	6	68	48	0.262449	2.896429	1.994582	3.464167	2.925978	Up	1.49E-05	1.38E-05	0.000146	0.000127	PTGS1
79799	3069	17	126	133	1.234012	8.906383	9.171474	2.851483	2.893797	Up	1.71E-08	1.38E-08	2.77E-07	2.07E-07	UGT2A3
84419	929	12	89	87	2.877616	20.7827	19.81928	2.852437	2.783959	Up	4.10E-07	3.53E-07	5.30E-06	4.27E-06	C15orf48
4547	4093	46	180	328	2.503706	9.540222	16.95963	1.929957	2.759968	Up	8.48E-14	4.42E-14	3.76E-12	1.63E-12	MTTP
6446	3208	9	73	64	0.624993	4.936467	4.222115	2.981566	2.756053	Up	2.59E-06	2.31E-06	2.93E-05	2.44E-05	SGK1
5243	4718	58	203	402	2.738655	9.333957	18.03236	1.769021	2.719048	Up	9.77E-15	0	5.08E-13	0	ABCB1
6692	2484	14	54	97	1.255578	4.715958	8.264271	1.909199	2.718536	Up	1.31E-06	9.76E-08	1.56E-05	1.28E-06	SPINT1
165679	2306	169	406	1164	16.32656	38.19394	106.8263	1.226123	2.709974	Up	7.77E-15	0	4.11E-13	0	C3orf57
11148	2689	74	353	508	6.130674	28.47814	39.98134	2.215737	2.705209	Up	2.11E-14	0	1.05E-12	0	HHLA2
7103	1159	82	262	555	15.76151	49.03945	101.343	1.637537	2.68477	Up	0	0	0	0	TSPAN8
10083	3246	60	322	404	4.117846	21.51964	26.34009	2.385693	2.677299	Up	0	0	0	0	USH1C
79762	2990	9	55	59	0.670562	3.990426	4.176046	2.573101	2.638696	Up	2.60E-06	2.32E-06	2.93E-05	2.44E-05	C1orf115
10158	894	18	125	117	4.485412	30.33194	27.697	2.757526	2.626417	Up	8.94E-09	7.14E-09	1.50E-07	1.11E-07	PDZK1IP1
2065	5765	150	468	964	5.796412	17.6106	35.3885	1.603212	2.610048	Up	9.55E-15	0	4.99E-13	0	ERBB3
3960	1291	250	820	1601	43.14009	137.7892	262.4518	1.675362	2.604951	Up	3.12E-13	1.39E-13	1.15E-11	4.60E-12	LGALS4
6564	3142	10	62	63	0.709024	4.280685	4.243447	2.593934	2.58133	Up	1.41E-06	1.25E-06	1.67E-05	1.38E-05	SLC15A1
6337	3497	74	245	464	4.71415	15.19841	28.08062	1.688851	2.574505	Up	2.11E-14	0	1.06E-12	0	SCNN1A
54825	4313	42	131	263	2.169388	6.589001	12.90508	1.602772	2.572579	Up	1.65E-11	7.28E-14	4.03E-10	2.61E-12	CDHR2
8876	3844	334	922	2086	19.35666	52.03257	114.8459	1.426585	2.568797	Up	3.32E-13	1.56E-13	1.21E-11	5.13E-12	VNN1
999	4815	45	199	280	2.082014	8.965706	12.30683	2.106438	2.563408	Up	2.62E-14	0	1.30E-12	0	CDH1
3672	4811	23	73	143	1.065025	3.291662	6.290499	1.627929	2.562287	Up	4.01E-07	2.59E-10	5.19E-06	4.88E-09	ITGA1
629	2646	11	60	68	0.926126	4.919139	5.438805	2.409125	2.554009	Up	7.67E-07	6.65E-07	9.48E-06	7.67E-06	CFB
9971	2492	18	124	110	1.609132	10.79447	9.341767	2.745938	2.537412	Up	8.94E-09	7.14E-09	1.49E-07	1.11E-07	NR1H4
8416	1843	11	58	67	1.329642	6.827007	7.693676	2.360216	2.532635	Up	7.72E-07	6.65E-07	9.53E-06	7.67E-06	ANXA9
10000	7091	11	49	66	0.345583	1.499051	1.969794	2.116944	2.51094	Up	1.54E-06	6.65E-07	1.80E-05	7.66E-06	AKT3
653190	2740	5	28	30	0.406525	2.216844	2.317155	2.447093	2.51094	Up	7.90E-05	4.94E-05	0.00066	0.000394	ABCC6P1
643008	4368	6	58	36	0.30601	2.880534	1.744232	3.234685	2.51094	Up	1.49E-05	1.74E-05	0.000146	0.000157	LOC643008
6374	2475	427	1196	2541	38.43439	104.8295	217.2771	1.447576	2.499066	Up	1.11E-12	8.62E-13	3.50E-11	2.38E-11	CXCL5
283120	2322	1481	8903	8532	142.0889	831.7674	777.63	2.549386	2.45229	Up	5.68E-12	4.85E-12	1.51E-10	1.15E-10	H19

140885	4201	47	199	263	2.49237	10.27609	13.24913	2.043702	2.410308	Up	0	0	0	0	SIRPA
5166	3710	7	35	39	0.420331	2.046548	2.224719	2.283594	2.404025	Up	2.17E-05	1.02E-05	0.000206	9.62E-05	PDK4
84699	2586	110	365	586	9.47614	30.61907	47.95716	1.692059	2.339375	Up	0	0	0	0	CREB3L3
8743	1953	9	37	47	1.026615	4.109862	5.093074	2.001195	2.310642	Up	3.93E-05	2.72E-06	0.000356	2.83E-05	TNFSF10
8537	3475	246	616	1268	15.77058	38.45507	77.22339	1.285938	2.291802	Up	3.14E-13	1.49E-13	1.15E-11	4.91E-12	BCAS1
2022	3196	9	215	45	0.62734	14.5935	2.979821	4.539934	2.247906	Up	2.59E-06	3.43E-06	2.93E-05	3.49E-05	ENG
56667	2899	109	285	524	8.376172	21.32673	38.25317	1.3483	2.191216	Up	0	0	0	0	MUC13
5507	2646	27	227	127	2.273219	18.61074	10.15777	3.033327	2.159775	Up	2.56E-11	1.84E-11	6.03E-10	3.99E-10	PPP1R3C
57530	5132	132	375	619	5.73	15.85157	25.52633	1.468019	2.155379	Up	1.46E-13	6.06E-14	5.99E-12	2.20E-12	CGN
397	1216	94	243	440	17.22113	43.35113	76.57791	1.33189	2.152749	Up	0	0	0	0	ARHGDIB
58191	2344	98	268	453	9.313991	24.80304	40.90016	1.413046	2.134635	Up	1.89E-14	0	9.64E-13	0	CXCL16
10863	3220	42	132	194	2.905767	8.892948	12.75059	1.613743	2.133573	Up	1.10E-11	7.28E-14	2.77E-10	2.62E-12	ADAM28
330	5243	211	745	970	8.965405	30.82507	39.15401	1.781664	2.126719	Up	2.99E-13	1.39E-13	1.11E-11	4.60E-12	BIRC3
5450	3032	53	123	224	3.894162	8.800425	15.63519	1.17626	2.005412	Up	2.51E-07	0	3.38E-06	0	POU2AF1
9536	1787	54	212	228	6.731883	25.73588	27.00192	1.934699	2.00398	Up	4.88E-14	0	2.28E-12	0	PTGES
58985	2988	14	60	59	1.043794	4.356105	4.178841	2.061202	2.001266	Up	1.87E-07	2.98E-07	2.58E-06	3.66E-06	IL22RA1
7348	2060	71	158	299	7.678183	16.63863	30.71768	1.1157	2.000232	Up	2.05E-08	0	3.28E-07	0	UPK1B
2243	3655	19	110	80	1.158067	6.528795	4.632197	2.495098	1.999978	Up	4.68E-09	4.99E-09	8.10E-08	7.98E-08	FGA
54658	2357	83	253	345	7.844872	23.28567	30.97733	1.56962	1.981391	Up	1.46E-13	8.30E-14	5.96E-12	2.92E-12	UGT1A1
54739	3638	12	62	49	0.734828	3.697062	2.850479	2.3309	1.955725	Up	4.13E-07	3.55E-06	5.34E-06	3.61E-05	XAF1
5176	1552	78	297	318	11.19619	41.5138	43.36305	1.890583	1.953458	Up	0	0	0	0	SERPINF1
1953	7450	29	119	118	0.86718	3.465121	3.352047	1.998503	1.95064	Up	7.29E-12	5.48E-12	1.89E-10	1.28E-10	MEGF6
140738	1687	29	94	117	3.829572	12.08761	14.6776	1.658274	1.938361	Up	5.31E-09	5.69E-12	9.17E-08	1.32E-10	TMEM37
10826	2997	21	65	83	1.560989	4.704943	5.861054	1.591717	1.9087	Up	2.57E-06	3.03E-09	2.92E-05	4.99E-08	C5orf4
84557	1030	17	52	65	3.676876	10.95201	13.35551	1.574643	1.860883	Up	3.12E-05	2.09E-07	0.000287	2.61E-06	MAP1LC3A
23576	4023	39	97	149	2.159643	5.230575	7.838278	1.276177	1.859744	Up	1.07E-06	7.97E-14	1.30E-05	2.82E-12	DDAH1
726	4393	148	700	564	7.505296	34.56723	27.17079	2.203424	1.856076	Up	0	0	0	0	CAPN5
90993	2702	86	254	326	7.090558	20.39276	25.53387	1.524086	1.848441	Up	0	0	0	0	CREB3L1
83648	4093	16	54	60	0.870854	2.862067	3.102372	1.716554	1.832868	Up	6.36E-06	7.87E-07	6.67E-05	8.96E-06	FAM167A
3552	2943	12	40	45	0.908361	2.948475	3.235986	1.698632	1.832868	Up	0.000121	2.00E-05	0.000964	0.000177	IL1A
56605	5070	10	42	37	0.439399	1.797087	1.544465	2.032056	1.813503	Up	1.00E-05	0.000128	0.000102	0.000931	ERO1LB
6318	1736	35	81	129	4.49144	10.12192	15.72622	1.172233	1.807922	Up	3.11E-05	7.12E-13	0.000286	2.01E-11	SERPINB4
55195	2512	14	46	51	1.241583	3.972518	4.296699	1.677873	1.791048	Up	4.25E-05	7.55E-06	0.000381	7.28E-05	C14orf105
619279	14403	92	285	335	1.422991	4.292592	4.922393	1.592922	1.790433	Up	1.98E-14	0	1.00E-12	0	ZNF704
219654	4933	23	64	82	1.038685	2.81447	3.51793	1.438104	1.759968	Up	1.49E-05	1.76E-08	0.000146	2.60E-07	ZCCHC24
2244	3628	27	242	96	1.657921	14.47024	5.600004	3.125642	1.756053	Up	2.56E-11	1.11E-09	6.04E-10	1.93E-08	FGB
22797	6631	38	179	134	1.27665	5.856009	4.276714	2.197555	1.744139	Up	1.25E-13	7.90E-13	5.23E-12	2.20E-11	TFEC

5337	5607	94	273	326	3.734777	10.56233	12.30471	1.499835	1.720117	Up	0	0	0	0	PLD1
11010	3924	262	608	894	14.87441	33.61261	48.21619	1.176171	1.696686	Up	1.44E-13	0	5.91E-12	0	GLIPR1
2185	4748	44	109	148	2.064473	4.980162	6.596832	1.270419	1.675999	Up	2.53E-07	2.59E-13	3.41E-06	8.19E-12	PTK2B
5740	5603	116	731	387	4.612163	28.30248	14.61756	2.617413	1.664186	Up	1.23E-13	4.62E-14	5.17E-12	1.70E-12	PTGIS
5973	1507	34	134	113	5.026122	19.28942	15.869	1.940293	1.658694	Up	4.41E-13	2.04E-10	1.54E-11	3.88E-09	RENBP
5794	3935	145	314	479	8.209006	17.31062	25.76174	1.076378	1.64995	Up	1.89E-14	0	9.68E-13	0	PTPRH
727910	5901	74	219	242	2.793659	8.050933	8.679089	1.527	1.635388	Up	2.11E-14	0	1.06E-12	0	TLCD2
1015	3699	893	2097	2890	53.78169	122.9821	165.3476	1.193261	1.620315	Up	0	0	0	0	CDH17
290	3740	277	1476	895	16.49968	85.61354	50.64491	2.375401	1.617979	Up	6.53E-14	0	2.96E-12	0	ANPEP
6999	1713	27	159	87	3.511347	20.13573	10.74846	2.519662	1.614034	Up	2.56E-11	4.67E-08	6.05E-10	6.43E-07	TDO2
23308	3239	23	62	73	1.581919	4.152489	4.769758	1.392301	1.59224	Up	3.21E-05	7.43E-07	0.000295	8.50E-06	ICOSLG
257629	2906	46	127	145	3.526384	9.4806	10.55983	1.426789	1.582325	Up	1.16E-09	3.21E-12	2.21E-08	7.89E-11	ANKS4B
9976	2052	46	124	143	4.993992	13.10907	14.74834	1.392301	1.562287	Up	3.57E-09	7.04E-12	6.27E-08	1.61E-10	CLEC2B
2635	3051	158	346	491	11.53672	24.6015	34.05836	1.092514	1.561776	Up	3.29E-14	0	1.59E-12	0	GBP3
3898	2869	154	434	478	11.95797	32.81609	35.25995	1.456431	1.560058	Up	2.15E-13	1.09E-13	8.41E-12	3.71E-12	LAD1
2770	3318	117	337	363	7.855554	22.03339	23.15339	1.487906	1.559439	Up	1.53E-13	8.19E-14	6.17E-12	2.89E-12	GNAI1
1396	480	33	127	102	15.31581	57.39713	44.97212	1.905957	1.554009	Up	7.88E-13	8.37E-09	2.59E-11	1.29E-07	CRIP1
5099	8728	141	457	434	3.598916	11.35871	10.52348	1.658165	1.547978	Up	6.88E-15	0	3.66E-13	0	PCDH7
11135	2182	72	212	221	7.350977	21.077	21.43492	1.519662	1.543955	Up	0	0	0	0	CDC42EP1
55450	2371	88	334	268	8.268342	30.55925	23.92146	1.885939	1.532635	Up	2.64E-14	0	1.31E-12	0	CAMK2N1
55711	2235	48	99	146	4.784439	9.609159	13.82483	1.00606	1.53084	Up	4.64E-05	8.70E-12	0.000411	1.97E-10	FAR2
27295	2853	64	211	194	4.997416	16.04384	14.39078	1.682765	1.525891	Up	0	0	0	0	PDLIM3
2678	2431	35	100	106	3.20738	8.923655	9.227952	1.476239	1.524615	Up	3.13E-08	6.94E-09	4.88E-07	1.08E-07	GGT1
83937	2509	44	93	133	3.906783	8.040999	11.21852	1.041393	1.521829	Up	4.91E-05	8.81E-11	0.000433	1.75E-09	RASSF4
3643	9059	150	310	451	3.688742	7.423507	10.53612	1.008972	1.514143	Up	4.37E-13	0	1.53E-11	0	INSR
1528	874	85	260	255	21.6658	64.53416	61.74661	1.574643	1.51094	Up	0	0	0	0	CYB5A
163175	2906	19	52	57	1.45655	3.88182	4.151104	1.414178	1.51094	Up	0.00012	2.65E-05	0.000953	0.000228	LGI4
1612	5942	166	551	497	6.223616	20.11623	17.70142	1.692535	1.50804	Up	2.16E-13	9.81E-14	8.39E-12	3.37E-12	DAPK1
23654	6369	574	1410	1707	20.07742	48.02591	56.72136	1.258239	1.498318	Up	6.76E-13	3.22E-13	2.26E-11	9.84E-12	PLXNB2
5465	10049	97	254	287	2.150385	5.483257	6.044264	1.350438	1.490972	Up	7.64E-14	1.49E-14	3.41E-12	5.74E-13	PPARA
1004	8571	365	1200	1075	9.486996	30.37228	26.54369	1.678732	1.484346	Up	7.03E-13	4.01E-13	2.34E-11	1.20E-11	CDH6
5920	779	32	76	94	9.151238	21.1643	25.53729	1.209594	1.480567	Up	3.55E-05	9.58E-08	0.000324	1.26E-06	RARRES3
79789	12750	116	339	340	2.02682	5.767893	5.64356	1.508827	1.477388	Up	1.23E-13	4.62E-14	5.18E-12	1.70E-12	CLMN
255394	2243	40	86	116	3.972812	8.317578	10.94493	1.066003	1.462031	Up	6.96E-05	4.18E-09	0.000592	6.76E-08	TCP11L2
1356	4674	897	2701	2581	42.75344	125.3613	116.8648	1.55198	1.450728	Up	5.44E-14	0	2.52E-12	0	CP
10344	562	153	408	439	60.64883	157.4895	165.3151	1.376704	1.446667	Up	0	0	0	0	CCL26
1520	4107	260	622	746	14.10314	32.85439	38.44134	1.220069	1.446642	Up	4.86E-13	2.99E-13	1.68E-11	9.22E-12	CTSS

5698	1048	20	66	57	4.25144	13.66188	11.5106	1.684132	1.43694	Up	8.09E-07	5.17E-05	9.93E-06	0.000412	PSMB9
345557	7739	30	110	85	0.863582	3.08344	2.324441	1.836135	1.428478	Up	1.54E-11	7.97E-07	3.80E-10	9.06E-06	PLCXD3
57104	2443	110	242	308	10.03082	21.48917	26.68159	1.09917	1.411405	Up	6.70E-12	0	1.75E-10	0	PNPLA2
50848	4830	249	668	694	11.4847	30.00247	30.40862	1.385369	1.404768	Up	4.84E-14	0	2.27E-12	0	F11R
1052	1269	69	159	192	12.11309	27.18086	32.0202	1.166025	1.402416	Up	5.62E-09	1.25E-13	9.66E-08	4.18E-12	CEBPD
10046	4608	32	102	89	1.547052	4.801925	4.087539	1.634092	1.401711	Up	1.75E-09	6.33E-07	3.25E-08	7.34E-06	MAMLD1
85315	4758	152	330	420	7.116828	15.04587	18.68139	1.080061	1.392296	Up	1.26E-13	7.99E-15	5.25E-12	3.13E-13	PAQR8
50486	978	45	110	124	10.2504	24.39953	26.83288	1.251173	1.388321	Up	3.09E-07	5.03E-09	4.09E-06	8.03E-08	G0S2
55200	3149	31	87	85	2.19309	5.993414	5.712559	1.450413	1.381172	Up	3.57E-07	1.49E-06	4.68E-06	1.63E-05	PLEKHG6
65268	6834	22	93	60	0.717158	2.952131	1.858064	2.041393	1.373437	Up	6.86E-10	5.93E-05	1.35E-08	0.000467	WNK2
54578	2495	55	126	150	4.910882	10.95539	12.72346	1.157586	1.373437	Up	2.55E-07	1.75E-10	3.43E-06	3.33E-09	UGT1A6
64856	4659	74	244	201	3.538395	11.36122	9.130357	1.68295	1.367576	Up	2.11E-14	1.34E-13	1.06E-12	4.47E-12	VWA1
10893	4344	195	488	529	10.00028	24.37012	25.77213	1.285073	1.365771	Up	2.37E-13	1.09E-13	9.12E-12	3.69E-12	MMP24
6237	1013	148	386	398	32.54765	82.66194	83.14919	1.34467	1.353149	Up	0	0	0	0	RRAS
3484	1660	103	218	274	13.82281	28.48893	34.93228	1.04335	1.337509	Up	4.30E-10	0	8.71E-09	0	IGFBP1
11118	4096	29	103	77	1.577268	5.455129	3.978462	1.790186	1.334783	Up	1.19E-10	8.21E-06	2.57E-09	7.86E-05	BTN3A2
8744	1680	22	58	58	2.917298	7.48939	7.306394	1.360216	1.324527	Up	7.85E-05	0.000123	0.000657	0.000902	TNFSF9
80221	2257	54	171	141	5.330028	16.43585	13.22123	1.624631	1.310642	Up	5.57E-14	2.39E-09	2.57E-12	3.98E-08	ACSF2
115294	4186	92	242	240	4.896164	12.54134	12.13379	1.356968	1.309306	Up	2.02E-14	0	1.02E-12	0	PCMTD1
51129	1967	91	221	237	10.30634	24.37337	25.49931	1.241774	1.306926	Up	3.77E-13	0	1.35E-11	0	ANGPTL4
6623	855	132	365	341	34.3934	92.60927	84.40587	1.429025	1.295212	Up	1.46E-13	6.06E-14	6.01E-12	2.21E-12	SNCG
80274	3885	59	197	152	3.383205	11.00026	8.280126	1.701075	1.291262	Up	3.33E-14	8.92E-10	1.60E-12	1.58E-08	SCUBE1
53841	3489	35	77	90	2.234778	4.787596	5.459161	1.09917	1.288548	Up	0.000115	2.65E-06	0.000921	2.76E-05	CDHR5
4035	14905	1086	2435	2777	16.23174	35.44008	39.43014	1.126564	1.280481	Up	3.64E-12	2.83E-12	1.02E-10	7.05E-11	LRP1
64116	4098	40	92	102	2.17448	4.870164	5.267598	1.1633	1.276475	Up	1.01E-05	6.81E-07	0.000102	7.83E-06	SLC39A8
3106	1578	31	124	79	4.37645	17.04678	10.59509	1.961666	1.275562	Up	2.18E-12	1.29E-05	6.43E-11	0.000118	HLA-B
54020	3098	83	188	211	5.968484	13.16449	14.41403	1.141216	1.272037	Up	4.61E-10	9.99E-13	9.32E-09	2.74E-11	SLC37A1
10347	6834	83	171	210	2.705643	5.428113	6.503224	1.004479	1.265184	Up	8.51E-08	1.37E-12	1.23E-06	3.64E-11	ABCA7
4241	3963	122	421	308	6.858088	23.04548	16.44792	1.748605	1.262027	Up	7.95E-14	0	3.54E-12	0	MFI2
694	4704	271	582	684	12.83421	26.84005	30.77324	1.064393	1.261681	Up	9.04E-14	0	3.96E-12	0	BTG1
64787	3156	193	421	487	13.62347	28.93829	32.65701	1.086886	1.261299	Up	1.21E-13	0	5.11E-12	0	EPS8L2
79776	13975	69	142	174	1.099929	2.204267	2.635007	1.002889	1.260397	Up	1.11E-06	1.22E-10	1.34E-05	2.37E-09	ZFHX4
3965	1835	35	87	88	4.249123	10.28516	10.14918	1.275327	1.256126	Up	3.93E-06	5.30E-06	4.28E-05	5.26E-05	LGALS9
2217	1893	75	171	188	8.826286	19.59626	21.01801	1.1507	1.251748	Up	2.21E-09	2.79E-11	4.03E-08	5.87E-10	FCGRT
715	2526	337	1092	837	29.72103	93.78146	70.12559	1.657819	1.238457	Up	0	0	0	0	C1R
84102	3548	148	341	362	9.292775	20.84964	21.59282	1.165841	1.21637	Up	0	0	0	0	SLC41A2
3990	1614	102	224	249	14.07875	30.10733	32.64978	1.096596	1.213554	Up	4.39E-11	0	1.01E-09	0	LIPC

3486	2638	378	1153	918	31.92158	94.81613	73.64653	1.570601	1.206086	Up	6.80E-13	3.67E-13	2.27E-11	1.11E-11	IGFBP3
5792	7733	440	964	1061	12.6757	27.04312	29.037	1.093196	1.195827	Up	0	0	0	0	PTPRF
23303	8774	93	205	224	2.361308	5.068552	5.402998	1.101988	1.194174	Up	2.48E-10	2.47E-12	5.20E-09	6.26E-11	KIF13B
3912	5866	907	2350	2179	34.4455	86.90675	78.61394	1.335153	1.190469	Up	0	0	0	0	LAMB1
3107	1543	120	312	288	17.32538	43.86482	39.50126	1.340178	1.189012	Up	0	0	0	0	HLA-C
768	1561	77	182	184	10.98892	25.29276	24.94591	1.202674	1.182753	Up	1.63E-10	3.03E-10	3.50E-09	5.65E-09	CA9
333	2450	51	187	121	4.637366	16.55782	10.4521	1.836135	1.172416	Up	1.47E-14	4.11E-07	7.54E-13	4.91E-06	APLP1
51257	1705	30	83	71	3.919803	10.56043	8.81289	1.429815	1.168834	Up	8.75E-07	0.000114	1.07E-05	0.000846	2-Mar
79629	1124	37	81	87	7.333355	15.63315	16.38088	1.092063	1.159468	Up	8.30E-05	2.15E-05	0.000689	0.000188	OCEL1
6648	1593	197	406	463	27.54976	55.2889	61.51055	1.00495	1.158794	Up	0	0	0	0	SOD2
11067	2060	80	194	188	8.651474	20.42971	19.31412	1.239651	1.158638	Up	1.37E-11	3.80E-10	3.42E-10	7.04E-09	C10orf10
1519	2943	86	295	200	6.509918	21.745	14.38216	1.739973	1.143569	Up	0	1.63E-10	0	3.12E-09	CTSO
435	2061	166	372	384	17.9431	39.15549	39.43099	1.125786	1.135901	Up	2.16E-13	9.81E-14	8.42E-12	3.38E-12	ASL
1028	1943	90	260	208	10.31899	29.02875	22.65557	1.492181	1.134564	Up	0	9.24E-11	0	1.83E-09	CDKN1C
54541	1752	153	394	348	19.45471	48.7854	42.03679	1.32633	1.111533	Up	0	0	0	0	DDIT4
8303	3307	107	256	243	7.208035	16.7932	15.55093	1.220199	1.109323	Up	0	5.89E-12	0	1.37E-10	SNN
340348	2900	132	376	296	10.14012	28.12662	21.60121	1.471861	1.091037	Up	1.46E-13	1.27E-13	5.97E-12	4.25E-12	TSPAN33
65258	2806	79	181	175	6.27201	13.99325	13.19881	1.157731	1.073408	Up	6.22E-10	1.35E-08	1.23E-08	2.02E-07	MPPE1
114990	2864	47	147	104	3.655882	11.13453	7.685015	1.60675	1.071829	Up	8.53E-13	1.27E-05	2.77E-11	0.000117	VASN
5365	6377	57	148	125	1.99125	5.034693	4.148375	1.33823	1.058872	Up	3.82E-10	2.11E-06	7.81E-09	2.25E-05	PLXNB3
1397	1236	57	140	125	10.27363	24.57182	21.40306	1.258059	1.058872	Up	6.47E-09	2.11E-06	1.10E-07	2.25E-05	CRIP2
221895	3210	47	100	103	3.261821	6.75807	6.790732	1.050934	1.057889	Up	2.21E-05	1.72E-05	0.000209	0.000155	JAZF1
4060	2116	96	234	210	10.10701	23.98987	21.00332	1.247068	1.055261	Up	0	8.30E-10	0	1.48E-08	LUM
1917	1841	200	519	437	24.20157	61.15631	50.23565	1.337401	1.053611	Up	0	0	0	0	EEF1A2
9572	2788	50	118	109	3.995256	9.18157	8.27405	1.200453	1.050306	Up	2.86E-07	1.10E-05	3.82E-06	0.000103	NR1D1
83872	18212	46	139	100	0.562688	1.655712	1.162055	1.557045	1.046272	Up	1.09E-11	2.71E-05	2.75E-10	0.000233	HMCN1
54504	1779	129	304	278	16.15404	37.07024	33.07145	1.198366	1.033692	Up	0	3.53E-12	0	8.59E-11	CPVL
780	3877	313	686	670	17.98522	38.38451	36.57324	1.093712	1.023976	Up	0	0	0	0	DDR1
54101	3890	72	186	153	4.12335	10.37268	8.323888	1.3309	1.013441	Up	2.62E-12	4.08E-07	7.61E-11	4.89E-06	RIPK4
975	1497	124	0	0	18.45301	0.001	0.001	-14.1716	-14.1716	Down	8.84E-39	1.83E-39	1.03E-36	1.27E-37	CD81
3575	1809	20	0	0	2.462968	0.001	0.001	-11.2662	-11.2662	Down	7.20E-07	5.53E-07	8.95E-06	6.47E-06	IL7R
5053	2680	25	1	0	2.078129	0.080946	0.001	-4.68219	-11.0211	Down	2.98E-07	1.52E-08	3.97E-06	2.26E-07	PAH
5396	4071	34	0	0	1.860566	0.001	0.001	-10.8615	-10.8615	Down	3.64E-11	2.34E-11	8.38E-10	4.99E-10	PRRX1
2925	2681	22	2	0	1.828072	0.161831	0.001	-3.49777	-10.8361	Down	1.46E-05	1.31E-07	0.000144	1.69E-06	GRPR
6769	2963	21	1	0	1.578901	0.073214	0.001	-4.43065	-10.6247	Down	4.31E-06	2.69E-07	4.66E-05	3.31E-06	STAC
55536	2976	20	2	0	1.497147	0.145789	0.001	-3.36026	-10.548	Down	5.11E-05	5.53E-07	0.000449	6.46E-06	CDCA7L
84969	2558	17	1	0	1.480525	0.084806	0.001	-4.1258	-10.5319	Down	6.07E-05	4.78E-06	0.000525	4.78E-05	TOX2

4037	3714	23	3	0	1.3796	0.175229	0.001	-2.97693	-10.43	Down		3.70E-05	6.39E-08	0.000336	8.64E-07	LRP3
26053	6426	21	1	0	0.728024	0.033759	0.001	-4.43065	-9.50784	Down		4.31E-06	2.69E-07	4.66E-05	3.31E-06	AUTS2
10814	4726	266	32	1	12.53878	1.468872	0.044781	-3.09362	-8.1293	Down		1.08E-48	1.13E-81	1.36E-46	9.23E-80	CPLX2
8549	2880	112	4	2	8.66349	0.301297	0.146968	-4.84569	-5.88138	Down		2.15E-29	1.79E-32	2.14E-27	1.16E-30	LGR5
54345	1718	45	16	1	5.835213	2.02034	0.123186	-1.53019	-5.56588	Down		0.000116	2.11E-13	0.00093	6.83E-12	SOX18
50964	2322	175	81	4	16.78971	7.567467	0.364571	-1.14969	-5.52523	Down		8.19E-10	6.45E-49	1.60E-08	4.73E-47	SOST
945	1466	29	0	1	4.406881	0.001	0.144361	-12.1055	-4.932	Down		1.25E-09	1.40E-08	2.36E-08	2.10E-07	CD33
3371	7616	54	2	2	1.579553	0.056968	0.055576	-4.79322	-4.82891	Down		1.13E-14	5.77E-15	5.84E-13	2.29E-13	TNC
85407	2604	110	6	5	9.410637	0.499848	0.406362	-4.23473	-4.53345	Down		9.74E-27	2.56E-28	8.80E-25	1.60E-26	NKD1
5521	2376	36	2	2	3.375386	0.182604	0.178143	-4.20826	-4.24395	Down		1.78E-09	1.14E-09	3.30E-08	1.97E-08	PPP2R2B
54967	2188	17	1	1	1.730888	0.099147	0.096725	-4.1258	-4.16149	Down		6.07E-05	4.89E-05	0.000525	0.000391	CXorf48
4313	3549	203	62	13	12.74258	3.789775	0.775214	-1.74947	-4.03892	Down		1.08E-19	1.84E-47	7.56E-18	1.33E-45	MMP2
5168	3276	131	24	13	8.908298	1.58926	0.839815	-2.48679	-3.40701	Down		6.70E-20	2.57E-27	4.72E-18	1.59E-25	ENPP2
3625	3218	251	24	26	17.37621	1.617905	1.709904	-3.42491	-3.34513	Down		1.88E-50	3.29E-50	2.47E-48	2.46E-48	INHBB
57159	2073	54	13	7	5.803123	1.360416	0.714633	-2.09278	-3.02155	Down		1.55E-07	6.90E-11	2.16E-06	1.38E-09	TRIM54
27345	1631	37	6	5	5.053766	0.798041	0.648784	-2.66282	-2.96155	Down		6.11E-07	1.05E-07	7.67E-06	1.37E-06	KCNMB4
2049	4234	43	13	6	2.262481	0.666071	0.299906	-1.76416	-2.91532	Down		3.07E-05	1.20E-08	0.000284	1.81E-07	EPHB3
59271	1977	52	13	8	5.859546	1.426476	0.856382	-2.03833	-2.77446	Down		4.20E-07	9.15E-10	5.42E-06	1.62E-08	C21orf63
9388	4143	52	6	8	2.79612	0.31417	0.408657	-3.15381	-2.77446	Down		9.32E-11	9.15E-10	2.04E-09	1.61E-08	LIPG
55959	4248	64	6	10	3.356316	0.306404	0.498196	-3.45337	-2.75209	Down		6.03E-14	1.22E-11	2.76E-12	2.70E-10	SULF2
860	5720	24	1	4	0.934722	0.037926	0.147995	-4.6233	-2.65898	Down		5.83E-07	5.95E-05	7.34E-06	0.000468	RUNX2
7474	5855	526	199	90	20.01364	7.373164	3.253119	-1.44063	-2.62109	Down		5.31E-37	4.71E-81	6.06E-35	3.83E-79	WNT5A
8840	5194	114	55	21	4.889565	2.297145	0.855661	-1.08986	-2.51459	Down		2.12E-06	5.81E-18	2.43E-05	2.62E-16	WISP1
56967	7504	108	53	20	3.206256	1.532183	0.564055	-1.0653	-2.50698	Down		5.90E-06	4.90E-17	6.21E-05	2.16E-15	C14orf132
4162	3332	48	11	9	3.20925	0.716169	0.571639	-2.16386	-2.48906	Down		4.51E-07	3.12E-08	5.77E-06	4.44E-07	MCAM
25825	2993	175	5	35	13.02563	0.362402	2.474832	-5.16762	-2.39595	Down		1.04E-46	2.04E-25	1.29E-44	1.19E-23	BACE2
1462	12416	862	59	186	15.46653	1.030856	3.170412	-3.90723	-2.28641	Down		########	########	########	########	VCAN
9315	2304	39	6	9	3.770939	0.564932	0.826693	-2.73877	-2.1895	Down		1.96E-07	4.06E-06	2.69E-06	4.10E-05	C5orf13
654	3105	179	51	42	12.84277	3.563168	2.862675	-1.84972	-2.16552	Down		8.08E-19	4.06E-23	5.38E-17	2.20E-21	BMP6
5522	4438	131	56	31	6.575841	2.737338	1.478287	-1.2644	-2.15325	Down		1.04E-08	3.39E-17	1.72E-07	1.50E-15	PPP2R2C
57407	1401	82	5	21	13.03896	0.774211	3.172237	-4.07396	-2.03926	Down		9.45E-20	1.29E-10	6.63E-18	2.51E-09	NMRAL1
85236	863	265	134	68	68.40729	33.68385	16.67564	-1.02209	-2.03641	Down		6.28E-12	4.56E-31	1.65E-10	2.93E-29	HIST1H2BK
219790	6659	42	10	11	1.405101	0.325776	0.349597	-2.10872	-2.00691	Down		3.54E-06	5.97E-06	3.88E-05	5.88E-05	RTKN2
51765	3352	299	149	80	19.87168	9.642951	5.050919	-1.04317	-1.9761	Down		1.13E-13	1.46E-33	4.79E-12	9.63E-32	MST4
3084	3220	214	109	59	14.80557	7.34342	3.877757	-1.01162	-1.93285	Down		9.23E-10	8.46E-24	1.79E-08	4.71E-22	NRG1
92935	3102	108	47	30	7.756205	3.286879	2.046746	-1.23863	-1.92202	Down		3.20E-07	1.22E-12	4.24E-06	3.27E-11	MARS2
347733	2019	126	43	35	13.90278	4.62019	3.668733	-1.58935	-1.92202	Down		2.54E-11	1.63E-14	6.00E-10	6.23E-13	TUBB2B

6909	3396	229	82	66	15.02225	5.238101	4.113018	-1.51999	-1.86883	Down		2.83E-18	2.65E-24	1.83E-16	1.51E-22	TBX2
493861	1447	64	15	19	9.853233	2.248798	2.778878	-2.13144	-1.82609	Down		7.16E-09	1.39E-07	1.21E-07	1.79E-06	EID3
83879	2839	200	89	61	15.69394	6.80068	4.54725	-1.20646	-1.78714	Down		8.86E-12	3.10E-20	2.27E-10	1.53E-18	CDCA7
6542	7584	94	5	30	2.761194	0.143021	0.837158	-4.27099	-1.72172	Down		3.75E-23	8.26E-10	3.04E-21	1.47E-08	SLC7A2
6402	2442	72	17	23	6.568318	1.510188	1.993272	-2.1208	-1.72039	Down		9.12E-10	8.18E-08	1.78E-08	1.09E-06	SELL
1896	5296	50	13	17	2.103243	0.532504	0.679337	-1.98175	-1.63042	Down		1.12E-06	1.78E-05	1.35E-05	0.00016	EDA
25907	1876	86	35	30	10.21252	4.047277	3.384331	-1.33532	-1.5934	Down		1.36E-06	2.79E-08	1.62E-05	3.99E-07	TMEM158
618	200	1154	565	410	1285.414	612.8387	433.8487	-1.06865	-1.56697	Down		3.71E-50	1.24E-90	4.80E-48	1.03E-88	BCYRN1
4133	9445	474	196	171	11.18005	4.501755	3.831586	-1.31237	-1.54491	Down		2.78E-29	1.68E-37	2.75E-27	1.13E-35	MAP2
164832	13920	268	127	97	4.289068	1.979211	1.474745	-1.11574	-1.5402	Down		1.13E-13	8.37E-22	4.82E-12	4.35E-20	LONRF2
4129	2611	329	47	125	28.0709	3.904979	10.13182	-2.84569	-1.47018	Down		4.03E-55	9.30E-25	5.39E-53	5.35E-23	MAOB
5327	3173	123	37	47	8.635796	2.529644	3.134817	-1.77139	-1.46195	Down		1.09E-12	4.31E-10	3.43E-11	7.91E-09	PLAT
8835	2210	114	18	44	11.49158	1.766884	4.213518	-2.7013	-1.44748	Down		4.67E-19	2.47E-09	3.18E-17	4.08E-08	SOCS2
22979	7500	82	23	32	2.435678	0.665264	0.90297	-1.87232	-1.43157	Down		1.67E-09	5.46E-07	3.12E-08	6.40E-06	EFR3B
2151	3427	278	136	109	18.07166	8.608996	6.731267	-1.06981	-1.42478	Down		2.71E-13	2.94E-20	1.02E-11	1.45E-18	F2RL2
3598	1376	635	299	254	102.807	47.13901	39.06607	-1.12494	-1.39595	Down		1.13E-30	6.44E-43	1.20E-28	4.60E-41	IL13RA2
54733	3184	266	118	109	18.61127	8.039641	7.244991	-1.21097	-1.36112	Down		2.86E-15	3.01E-18	1.58E-13	1.37E-16	SLC35F2
6713	2989	807	359	333	60.14713	26.05531	23.57777	-1.20692	-1.35107	Down		6.54E-43	1.59E-51	7.87E-41	1.20E-49	SQLE
149175	2858	107	43	46	8.340438	3.263878	3.406277	-1.35354	-1.29193	Down		5.04E-08	1.19E-07	7.63E-07	1.54E-06	MANEAL
586	9682	96	9	42	2.208887	0.201653	0.918055	-3.45337	-1.26667	Down		2.73E-20	7.91E-07	1.96E-18	9.00E-06	BCAT1
3855	1753	113	28	50	14.36031	3.465005	6.036323	-2.05116	-1.25035	Down		5.13E-14	1.12E-07	2.39E-12	1.46E-06	KRT7
1678	1459	110	51	49	16.79596	7.583027	7.107636	-1.14727	-1.24067	Down		1.21E-06	1.96E-07	1.45E-05	2.46E-06	TIMM8A
6926	4814	197	65	88	9.116486	2.929105	3.868664	-1.63802	-1.23664	Down		1.39E-17	3.53E-12	8.71E-16	8.59E-11	TBX3
51655	1814	122	9	55	14.98269	1.076299	6.416672	-3.79915	-1.2234	Down		2.18E-27	5.93E-08	2.05E-25	8.07E-07	RASD1
56654	1548	77	37	35	11.08121	5.185116	4.784995	-1.09567	-1.21153	Down		9.31E-05	1.99E-05	0.000765	0.000176	NPDC1
4680	2631	3876	501	1764	328.1937	41.30899	141.8934	-2.99002	-1.20974	Down		0	########	0	########	CEACAM6
57214	7080	190	37	87	5.978437	1.133695	2.600581	-2.39874	-1.20093	Down		4.18E-27	2.50E-11	3.89E-25	5.29E-10	KIAA1199
9467	2810	138	57	64	10.94057	4.400441	4.820122	-1.31397	-1.18255	Down		1.41E-09	1.98E-08	2.65E-08	2.89E-07	SH3BP5
4953	2062	310	140	145	33.49194	14.72879	14.88208	-1.18518	-1.17024	Down		5.19E-17	6.47E-17	3.17E-15	2.82E-15	ODC1
26227	2021	134	30	63	14.77086	3.220199	6.597185	-2.19753	-1.16283	Down		1.01E-17	4.82E-08	6.36E-16	6.61E-07	PHGDH
29015	2867	79	17	38	6.138563	1.28632	2.805048	-2.25465	-1.12988	Down		2.56E-11	4.35E-05	6.05E-10	0.000354	SLC43A3
23507	7823	163	79	79	4.641748	2.190693	2.137166	-1.08328	-1.11897	Down		1.62E-08	5.24E-09	2.64E-07	8.32E-08	LRRC8B
8313	4241	553	282	270	29.04853	14.42476	13.47348	-1.00992	-1.10834	Down		7.72E-23	1.15E-26	6.16E-21	6.89E-25	AXIN2
84133	6696	250	123	124	8.317482	3.9849	3.919139	-1.0616	-1.08561	Down		5.74E-12	1.65E-12	1.52E-10	4.33E-11	ZNRF3
2810	1336	408	184	205	68.03322	29.87714	32.4737	-1.1872	-1.06697	Down		5.83E-22	5.62E-19	4.49E-20	2.67E-17	SFN
23276	4743	186	93	94	8.736292	4.253609	4.194297	-1.03833	-1.05859	Down		5.55E-09	2.46E-09	9.55E-08	4.07E-08	KLHL18
284098	2255	104	49	53	10.27434	4.713866	4.974091	-1.12406	-1.04654	Down		3.45E-06	1.02E-05	3.79E-05	9.63E-05	PIGW

2180	3822	652	325	336	38.00356	18.44677	18.60514	-1.04277	-1.03043	Down		5.41E-28	7.71E-28	5.19E-26	4.78E-26	ACSL1
6549	5446	191	80	100	7.813094	3.186692	3.886035	-1.29383	-1.00759	Down		1.87E-12	6.48E-09	5.58E-11	1.02E-07	SLC9A2

Supplementary Table S3

MiRNA Target clone	
**ERBB3 3'UTR site**	**Mutant Sequence**
822-829bp	agcacttt **mutate into** tcgtgaaa
189-195bp	gcacttt **mutate into** cgtgaaa
664-671bp	agcacttt **mutate into** tcgtgaaa

Supplementary Table S4. Correlation between ERBB3 expression and clinicopathologic in HCC patients

Characteristics	Cases (*N*=104)	Relative ERBB3 expression		*P*-value*
		Low (*N*=30)	High (*N*=74)	
Sex				0.108
Male	86	22	64	
Female	18	8	10	
Age (year)				0.808
≤50	47	13	34	
>50	57	17	40	
Liver cirrhosis				0.715
Yes	12	4	8	
No	92	26	66	
AFP (ng/ml)				**0.023**
≤20	35	16	19	
>20	68	14	54	
NA	1	0	1	
GGT (U/L)				**0.012**
≤54	47	20	27	
>54	55	9	46	
NA	2	1	1	
ALT(U/L)				0.089
≤75	87	28	59	
>75	17	2	15	
HBsAg				0.432
Positive	87	27	60	
Negative	14	2	12	
NA	3	1	2	
HCVAb				0.936
Positive	9	3	6	
Negative	92	26	66	
NA	3	30	74	
Tumor size (cm)				**0.011**
≤5cm	56	22	34	
>5cm	48	8	40	

Tumor number				0.165
Single	85	27	58	
Multiple	19	3	16	
Tumor encapsulation				0.423
Complete	56	18	38	
None	48	12	36	
Tumor differentiation				
I+II	76	22	54	0.069
III+IV	26	6	20	
NA	2	2	0	
Tumor thrombus				**0.047**
Yes	32	5	27	
No	72	25	47	
TNM stage				**0.007**
I	70	26	44	
II+III	34	4	30	
MiR-17-5p				**0.000**
Low	72	6	66	
High	32	24	8	
MiR-20a-5p				**0.000**
Low	70	6	64	
High	34	24	10	

Numerals labeled in boldface if P value <0.05. Chi-square tests for all analyses.
Abbreviations: AFP, alpha-fetoprotein; GGT, gamma glutamyl transferase; TNM, tumor node-metastasis; NA, not available.

Supplementary Table S5

**ERBB3 promoter PCR primer**	
**Primer No.**	**Sequence (5'-3')**
ERBB3 site2+	AAACAAATAAACAAAGTAGCCAGACA
ERBB3 site2-	CTGACCTCAGGTGATCCACCCAC
ERBB3 site1+	GGGAGGTTCCAGAGGTCCACGCC
ERBB3 site1-	ACCTGTCGCCGTCCCCTCTGCTG

Supplementary Table S6

**PCR primer**	
**Primer**	**Sequence (5'-3')**
ERBB3-F	GGACAGTACGGGAGATCACAG
ERBB3-R	GCACTAATTTCCTTCAGGGATCG
GAPDH-F	GAAGGTGAAGGTCGGAGT
GAPDH-R	GAAGATGGTGATGGGATTTC

Supplementary Table S7

ERBB3 shRNA site	
**ID**	**Target Sequence (5’-3’)**
ERBB3-RNAi(15932)	CAAGGGAATGTACTACCTT
ERBB3-RNAi(18869)	TGACAAGATGGAAGTAGAT

Supplementary Materials and Methods

Isolation and sequencing of small RNA

RNA sequencing was performed according to the manufacturer's instructions. In brief, total RNA was isolated from HCCLM3R, HCCLM3R-LM1-S4 and HCCLM3R-LnM1-S11 with Trizol reagent (Invitrogen, Grand Island, NY) and quantitated by 2100-Bioanalyzer (Agilent Technologies, Santa Clara, CA). High quality RNA (1 μg) was used to build a small RNA library. The Truseq small RNA sample preparation kit was used for 30 adaptors ligation, 50 adaptors ligation, reverse transcription, PCR amplification, and polyacrylamide gel electrophoresis purified small RNA library. RNA sequencing was performed using HiSeq2000 (Illumina Inc., San Diego, CA) at the Beijing Genomics Institute (Beijing, China). Then, the adapter sequences were removed by a FASTXTool kit (0.0.14). After adaptor trimming, reads were aligned to the SOAPaligner/soap2. The normalized expression value of each mRNA was calculated as the RPKM (Reads Per Kb per Million reads). The differentially expressed genes (DEGs) were selected by p value< 0.05 and the absolute value of log2 fold-change ≥ 1.

Real-time PCR used for miRNA and mRNA assays

Total RNAs were extracted from cultured cells with Trizol Reagent (Thermo Fisher Scientific, Waltham, MA, USA). The quality and integrity of RNA were evaluated via A260/A280 ratio, and then 1 μg of

total RNA was used for first-strand DNA synthesis. Real-time PCR was performed in triplicate by the SYBR Green PCR method using an All-in-One miRNA qPCR Detection kit (GeneCopoeia, Rockville, MD, USA). The forward primers of hsa-miR-17-3p, hsa-miR-17-5p, hsa-miR-18a-5p, hsa-miR-19a-3p, hsa-miR-19b-3p, hsa-miR-20a-5p， hsa-miR-92a-1-5p, the common reverse primer and U6 small nuclear RNA (U6) were synthesized by GeneCopoeia. For mRNA detection, 1 μg of total RNA was used for complementary DNA synthesis with a PrimeScript RT reagent kit (Takara Bio, Kyoto, Japan). Real-time PCR was performed in triplicate using SYBR Premix Ex Taq (Takara Bio， Japan). The primers for ERBB3 were synthesized by GenePharma (GenePharma, Shanghai, China) as Supplementary Table S6. The U6 and GAPDH were used as internal control for miRNAs and mRNAs assays, respectively. The threshold cycle (Ct) values were analyzed using the comparative Ct (−ΔCt) method. The levels of targets were obtained after normalized with endogenous reference and relative control.

Oligonucleotides, vectors and transfections

miR-17-5p and miR-20a-5p inhibitor, mimic and irrelevant oligonucleotides (NC) (Thermo Fisher Scientific, OH, USA) were used for transiently function observations in this study. And two GV248 plasmid particles containing short hairpin sequences against human ERBB3 gene as well as blank plasmids were purchased from GeneChem

(GeneChem, Shanghai, China). All these oligonucleotides and vectors were transfected into HCC cells using Lipofectamine 2000 (Thermo Fisher Scientific, MA, USA). The sequences of two shRNAs were shown in Supplementary Table S7. Ubi-Luc-MCS-IRES-Puromycin-miR-17-5p/miR-20a-5p overexpression lentiviruses, mU6-MCS-Ubi-Luc-miR-17-5p/miR-20a-5p knockdown lentiviruses and their corresponding negative control lentiviruses were purchased from Shanghai GeneChem Co. The miR-17-5p and miR-20a-5p overexpression lentiviruses were infected into HCCLM3 and the knockdown lentiviruses into HepG2. Ubi-MCS-Luc-IRES-Puromycin-ERBB3 and -HGF overexpression lentiviruses and its negative control lentiviruses were infected into HCCLM3 cells. Stably expressed clones were selected by qRT-PCR and immunoblotting assays.

Protein levels detected by Western blot assays

Lysates were obtained from cultured cells with a mixture of ProteoJET Mammalian Cell Lysis Reagent (Fermentas, MA, USA) and phenylmethanesulfonyl fluoride (Roche, Basel, Switzerland) and PhosSTOP (Roche). Proteins were separated by sodium dodecyl sulfate-PAGE and transferred onto polyvinylidene fluoride membranes. After blocked with 5% nonfat milk or bovine serum albumin, antibodies

against human ERBB3 (1:1,000; CST, Cell Signaling Technology), phospho-ERBB3 (1:1,000; Abcam), AKT (1:1,000; CST), phospho-AKT (1:1,000; CST), Erk (1:2,000; CST), phospho-Erk (1:1,000; CST), E-cadherin (1:1,000; CST), Vimentin (1:1,000; CST), NF-κB (1:1,000; CST), Twist (1:1,000; CST), Snail (1:1,000; CST), PI3K (1 : 1000; ProteinTech Group, Chicago, IL, USA), and GAPDH (1:10,000; Abcam) were probed and incubated with goat anti-rabbit or anti-mouse IgG(1:10,000 for both; Jackson ImmunoResearch Laboratories) respectively, then detected with enhanced chemiluminescence reagents (Thermo Fisher Scientific). The bands were visualized using 1-stepTM NBT/BCIP reagents (Thermo Fisher Scientific) and detected by Tanon 5200 automatic chemiluminescence imaging system (Tanon, Shanghai, China).

In vitro migration and invasion assays

Cell migration and invasion were analyzed by a Transwell Permeable Supports system with 8-μm pores (Corning, NY, USA). For migration assays, 5×10^4 cells were seeded into upper uncoated inserts. For invasion assays, 1.0×10^5 cells were seeded into upper inserts with a Matrigel-coated membrane (BD, NJ, USA). Cells were seeded in 1% serum medium and translocated to 10% serum media for 24 or 48 h. After removals of non-migrating or non-invading cells, the remaining cells were fixed, stained, and then cells were imaged and counted in at

least 5 random fields under an inverted microscopy (Olympus, Tokyo, Japan). All tested cells were pretreated either with 80 nM of miR-17-5p or miR-20a-5p inhibitors, mimics and irrelevant oligonucleotides for 60 h, or with 0 ng/ml，20 ng/ml and 40 ng/ml HGF for 1 h, or stably infected with ERBB3-shRNA (60 nM) and -overexpressed vector (80 nM) before plating.

Luciferase reporter assay

The binding sites for miR-17-5p and miR-20a-5p in 3'UTR sequence of ERBB3 were cloned into the pmirGLO, and the full sequence of ERBB3 promoter with NF-κB binding sites were cloned into pGL4.10 GLO Dual-Luciferase Expression Vector (Promega, WI, USA). Wild-type constructs, mutants and control vectors were co-transfected into target cells in 24-well plates together with miR-17-5p or miR-20a-5p mimics or inhibitors or NF-κB overexpressed plasmid using Lipofectamine 2000. Luciferase activities were measured 24 h after transfection using the Dual-Luciferase Reporter Assay System (Promega). The levels of firefly luciferase activities were obtained by normalizing to Renilla luciferase activities and relative to a control.

Immunohistochemical staining

Immunohistochemistry for ERBB3 was performed on 4 μm thick tissue microarray (TMA) made from 104 HCC surgical FFPE samples. The slides were probed with a primary antibody against ERBB3 (1:200;

CST), and then incubated with horseradish peroxidase-conjugated IgG (1:500; Thermo Fisher Scientific), and the proteins in situ were visualized with 3, 3’-diaminobenzidine. The intensity of positive staining was measured with integrated optical density (IOD) twice independently. In detail, the density of all stained chip were measured by a computerized image system, Leica DM IRE2 microscope(Leica Microsystems Imaging Solutions Ltd, Cambridge, United Kingdom) and photographed under 200 magnified visual field in the same parameters. Image-proplus v6.0 software (Media Cybernetics Inc, Bethesda, MD) was used for analysis. The typical tan color was used as the positive staining of target protein. IOD value was calculated according to the positive staining intensity and area. Staining intensity of target protein also named mean IOD (mIOD) was acquired through dividing by the area of tissue.

MiRNA in situ hybridization

MiRNA fluorescence in situ hybridization (FISH) assays were performed with 5’-cy3-labeled probes for mature human miRNAs (GenePharma, Shanghai, China). The following miRNA probes were used: miR-17-5p, 5’-C+TACCTGCAC+TGTAAGCACT+TTG-3’; miR-20a-5p, 5’-C+TACCTGCACT+ATAAGCACTTT+A-3’. FISH was performed on TMA of FFPE tissue. Briefly, slides were dewaxed and rehydrated rehydrated with graded alcohol washes. After washed with

RNase-free PBS, slides were digested with 15 μg/ml proteinase K at 37°C for 5 minutes. Then, sections were prehybridized in hybridization solution for 30 minutes at 37°C. Hybridization was performed with 7.5μl probes and 100μl hybridization solution at 37°C overnight. After stringency washes (4×, 2×, 1 × SSC) at 42°C, nuclei of cells were stained with DAPI. All the procedures were conducted according to the manufactory's instruction (GenePharma, Shanghai, China). Tissue sections were independently examined by two experienced pathologists using an Olympus IX73 microscope (Olympus, Tokyo, Japan). The average of the two scores was calculated. The expression level of miRNA was presented as 0, 1+, 2+ and 3+ for negative, weak, intermediate and strong signal respectively (Figure 1E). miRNA high expression was defined as scores 2-3 and miRNA low expression was defined as scores 0-1.

Enzyme-linked immunosorbent assay（ELISA）

The levels of cytokines in culture supernatant were determined by Quantikine Human ELISA kit (R&D Systems，MN，USA) as usually. Briefly, 100 μl samples were added to each well of the plates and incubated for 2.5 h at RT. The plates were washed and incubated with the conjugate for 2 h. After adding substrate solution, the absorbance was determined using a Microplate Spectrophotometer (Bio-Rad, CA, USA). The concentrations of samples were plotted by the absorbance curve of

standard cytokines/chemokines.

Proximity ligation assay (PLA)

Cells were seeded at density of 3 × 10^4 cells/slide, fixed with 4% paraformaldehyde for 10 minutes at room temperature and blocked with prepared solution (Duolink® In Situ, Sigma) for 30 minutes at 37°C on the next day. The slides were immunostained at 4°C overnight with anti-ERBB3 (1:50) and anti-MET (1:250) antibodies, probed with PLA solution (Duolink® In Situ, Sigma), ligated by ligation-ligase solution (Duolink® In Situ, Sigma) for 1 hour at 37°C and then amplified by polymerase solution (Duolink® In Situ, Sigma) at 37°C for 100 minutes. The visual spot was determined by a fluorescence microscope.

Chromatin immunoprecipitation (ChIP)

Cells were fixed with 37% formadehyde for 15 minutes at room temperature, stopped by 1M glycine and washed with ice-cold PBS twice. Then cells were scraped off, lysed with 1ml ChIP buffer (Millipore, MA, USA) and sonicated at 30 Amp for total 3 minutes. After quantification, 1mg lysates were immunopreciated with indicated antibody or control IgG at 4°C overnight. The immunoprecipitated DNAs containing NF-κB-binding site were amplified by PCR with ten primers against ERBB3 promoter (Supplementary Table S5) and sequenced.

Immunofluorescence staining

HCC cells were re-suspended in fresh medium, and adjusted to a

concentration of 1×104 cells/10μl into a 24-well glass dish. Monolayer cells grew into 70%-80% confluency, and then were fixed with 4% paraformaldehyde. Next, the cells were incubated with Vimentin and E-Cadherin antibody (1: 200) in 4℃ overnight followed by Alexa Fluor 488-donkey anti-rabbit IgG (1: 250; ProteinTech Group, Hubei, China) and Alexa Fluor 594-conjugated goat anti-mouse IgG (1: 250; ProteinTech Group) for 1h in the dark. The cell nuclei were counterstained with DAPI (Sangon Biotech, Ltd). Images were obtained under the Laser Scanning Confocal Microscope-TCS SP5 (Leica, Heidelberg, Germany).

Co-Immunoprecipitation

Firstly, add ice-cold lysis buffer to cell culture dish, Scrape adherent cells off the dish then transfer into a new tube. Constant agitation should be maintained for 30 minutes and centrifuge to collect the supernatant. Add about 50 μL off-target antibody and incubate for 1 hour at 4°C in order to pre-clearing the lysate, help reduce non-specific binding and reduce background. After that, quantify the sample protein and add 10μg ERBB3 monoclonal antibody into 500μg sample, incubating overnight at 4°C under gentle rotation. Next, Mix the slurry well and add 70–100μL of protein G-coupled Sepharose beads to each sample for 2 hours incubation. Next, wash the beads with washing buffer for three times to remove non-specific binding, centrifuge at 4°C and discard the

supernatant. Then, elute with 50 μL 2 x SDS buffer containing DTT. At last, boil the eluted samples for 5 minutes and analyze content of the sample by western blot. Of note, all the above steps were done on ice.
